# *In vitro* selection of miltefosine resistance in promastigotes of *Leishmania donovani* from Nepal: genomic and metabolomic characterization

**DOI:** 10.1111/mmi.13291

**Published:** 2016-02-09

**Authors:** C. D. Shaw, J. Lonchamp, T. Downing, H. Imamura, T. M. Freeman, J. A. Cotton, M. Sanders, G. Blackburn, J. C. Dujardin, S. Rijal, B. Khanal, C. J. R. Illingworth, G. H. Coombs, K. C. Carter

**Affiliations:** ^1^ Strathclyde Institute of Pharmacy and Biomedical Sciences, University of Strathclyde161 Cathedral StreetGlasgowG4 0REUK; ^2^ Wellcome Trust Sanger Institute, Wellcome Trust Genome CampusCambridgeUK; ^3^ College of Science, NUI GalwayGalwayIreland; ^4^ Department of Biomedical SciencesInstituut voor Tropische Geneeskunde NationalestraatAntwerpenBelgium; ^5^ Department of GeneticsUniversity of CambridgeCambridgeUK; ^6^ Department of Biomedical SciencesUniversity of AntwerpUniversiteitsplein 1AntwerpenBelgium; ^7^ BP Koirala Institute of Health SciencesDharanNepal; ^8^ Glasgow Polyomics, University of GlasgowGlasgow; ^9^ Present address: School of Biotechnology, Dublin City UniversityDublinIreland

## Abstract

In this study, we followed the genomic, lipidomic and metabolomic changes associated with the selection of miltefosine (MIL) resistance in two clinically derived *Leishmania donovani* strains with different inherent resistance to antimonial drugs (antimony sensitive strain Sb‐S; and antimony resistant Sb‐R). MIL‐R was easily induced in both strains using the promastigote‐stage, but a significant increase in MIL‐R in the intracellular amastigote compared to the corresponding wild‐type did not occur until promastigotes had adapted to 12.2 μM MIL. A variety of common and strain‐specific genetic changes were discovered in MIL‐adapted parasites, including deletions at the LdMT transporter gene, single‐base mutations and changes in somy. The most obvious lipid changes in MIL‐R promastigotes occurred to phosphatidylcholines and lysophosphatidylcholines and results indicate that the Kennedy pathway is involved in MIL resistance. The inherent Sb resistance of the parasite had an impact on the changes that occurred in MIL‐R parasites, with more genetic changes occurring in Sb‐R compared with Sb‐S parasites. Initial interpretation of the changes identified in this study does not support synergies with Sb‐R in the mechanisms of MIL resistance, though this requires an enhanced understanding of the parasite's biochemical pathways and how they are genetically regulated to be verified fully.

## Introduction

Visceral leishmaniasis (VL, also called kala‐azar) is a neglected tropical disease responsible for at least 40,000 deaths/year, the majority of which occur in the Indian sub‐continent (ISC, Alvar *et al*., 2012). There are a limited number of drugs available for chemotherapy of VL and development of drug resistance limits their clinical efficacy (Ready, 2014). Understanding the molecular mechanisms responsible for drug resistance may allow (i) the development of assays to predict the drug susceptibility of a clinical isolate, (ii) the design of interventions to extend the clinical life of a drug, and (iii) the guiding of R&D of new drugs.

First‐line chemotherapy in the ISC has relied on pentavalent antimonials since 1923 (among others, Sodium Stibogluconate or Meglumine Antimoniate). Antimonials have now been abandoned in the region because of decreased clinical efficacy and high resistance rates of the etiological agent, *Leishmania donovani* (Ready, 2014). Antimony‐resistant (Sb‐R) strains are still frequently found in patients of the ISC, despite a lower antimonial pressure in the region since its replacement by miltefosine (MIL, hexadecylphosphocholine): this could be explained by the greater virulence of Sb‐R parasites (Vanaerschot *et al*., 2014). Thus Sb‐R parasites are more likely to survive in the host and be transmitted to a new host because they are more resistant to the effectors of macrophages than Sb‐S parasites (Carter *et al*., 2005; Mukhopadhyay *et al*., 2011; Mukherjee *et al*., 2013; Guha *et al*., 2014; Vanaerschot *et al*., 2014). MIL was registered in India in 2002 and it is the first oral drug for VL treatment. It is one of the main pillars of the current kala‐azar elimination programme that aims to reduce the incidence of the disease in the ISC to lower than one case per 10,000 individuals at district or sub‐district levels by 2015 (WHO‐SEARO, 2011). After 10 years of use, the efficacy of MIL is decreasing in India (Sundar *et al*., 2012) and Nepal (Rijal *et al*., 2013). Fully MIL‐resistant (MIL‐R) strains have not yet been encountered in the ISC (Rijal *et al*., 2013; Prajapati *et al*., 2013) but this is probably only a matter of time, as parasites with varying MIL susceptibilities have been reported (Bhandari *et al*., 2012).

In the absence of MIL‐R clinical isolates, the primary way to study the mechanisms of resistance and to find molecular markers is through experimental selection. Previous reports showed that *Leishmania* rapidly develop MIL resistance in experimental conditions (Pérez‐Victoria *et al*., 2003) and that it was associated with a decreased accumulation of the drug. Two mechanisms were involved: (i) an increased drug efflux mediated by the overexpression of an ATP‐binding cassette (ABC) transporter P‐glycoprotein (LdBPK_341060, ABCB4), and (ii) a decreased uptake through the inactivation of a P‐type ATPase, the MIL transporter LdMT (LdBPK_131590) and its beta subunit LdRos3 (Pérez‐Victoria *et al*., 2006). More recently, a series of high‐throughput molecular screening methods were applied to scrutinize adaptations developed in selected MIL‐R parasites. Genomic analysis of MIL‐R *Leishmania major* confirmed the importance of the P‐type ATPase and identified mutations in other genes (Coelho *et al*., 2012). In another study, RNA microarray investigation detected many differentially expressed genes in MIL‐R *L. donovani*, highlighting a compromised DNA replication/repair mechanism, reduced protein synthesis and degradation, increased drug efflux, altered energy utilization and increased antioxidant defence mechanisms (Kulshrestha *et al*., 2014). Finally, the metabolomic analysis of an Ethiopian MIL‐R *L. donovani* strain highlighted an increase in the levels of amino acids, possibly to promote the adaptation of MIL‐R strains in the macrophage (Canuto *et al*., 2014).

Earlier work on MIL‐resistance has focused on laboratory strains sampled from patients decades ago. This does not take into account previous adaptations to other drugs, in particular antimonials, which have exerted continuous selective pressure on natural populations of *L. donovani* in the ISC since 1923 (Downing *et al*., 2011; Decuypere *et al*., 2012). Consequently, we experimentally selected MIL‐resistance in an Sb‐R and Sb‐S *L. donovani* strain recently derived from clinical isolates of Nepalese patients. To comprehensively evaluate the underlying mechanisms associated with resistance to MIL, we compared the genome, metabolome and lipidome of wild‐type (WT) strains and lines gradually adapted to MIL.

## Results

### Genomic differences between WT Sb‐S and Sb‐R strains prior to MIL exposure

High‐depth genome sequence data allowed the identification of key genomic differences between the two wild type (WT, summarized in Table 1) *L. donovani* isolates prior to adaption to MIL (further referred to as WT Sb‐R and WT Sb‐S). Some of these were potentially associated with antimonial resistance in the Sb‐R. Firstly, eight changes in chromosome copy number distinguished the pair: the WT Sb‐R had a higher dose of chromosomes 2, 8, 11, 14 and 33 and a lower copy number of chromosomes 5, 26 and 35 (Supporting Information Table S2). Secondly, there were 127 SNPs between the two isolates (Supporting Information Table S3), including 33 non‐synonymous single‐nucleotide polymorphisms (SNPs, Supporting Information Table S4A), of which 10 were previously associated with Sb‐R in the ISC (Downing *et al*., 2011). Thirdly, 16 loci had a local copy number variation (CNV), affecting 30 genes and 3 non‐coding regions (Supporting Information Table S4B). Fourthly, 38 deletions and 13 insertions differentiated the pair, including a deletion in the WT Sb‐R of two bases at the AQP1 gene (aquaglyceroporin 1, LdBPK_310030, Supporting Information Table S4C).

**Table 1:** Summary of the genomic differences observed between Sb‐S and Sb‐R strains during the selection of MIL‐resistance. Somy values are rounded to the nearest unit.

Standing variation at outset	Somy^a^	SNPs^b^	CNVs^c^	Indels^d^
WT Sb‐S vs WT Sb‐R differences	8	127	16	51
Above affecting amino acids		33	13	33
Effect of MIL on above differences	Yes in 5/8	none	none	None
New mutations during MIL‐R	Somy^a^	SNPs^e^	CNVs^f^	Indels
Identical in Sb‐S and Sb‐R^g^	Lower chr13	none	none	None
At same gene in Sb‐S and Sb‐R		A691P in Sb‐S, E197D in Sb‐R	ΔLdMT in Sb‐S	None
Unique to Sb‐S	2	1 hom^h^ + 17 het	8 (8 CDS)	None
Unique to Sb‐R	4	6 hom^i^ + 7 het	8 (298 CDS)	None

For full data see Tables: ^a^S2, ^b^S3 and S4A, ^c^S4B, ^d^S4C, ^e^S7 and S8, and ^f^S10. ^g^Chr8 and chr33 also had reduced copy numbers. ^h^A691P. ^i^Includes E197D.

### Differences in metabolite and lipid profile between WT strains prior to MIL exposure

Approximately 350 metabolites were annotated with IDEOM, 14 of them significantly different between the two WT strains (summarized in Table 2, *P*‐value < 0.05). These metabolites were associated with different metabolic pathways including protein production, lipid metabolism and synthesis of DNA. Over 400 lipids were seen in promastigote samples but only 21 were significantly different between the two strains (*P* < 0.05, Supporting Information Table S6). Sb‐S WT promastigotes had significantly higher levels of 10 phosphatidylcholines (PC) and significantly higher levels of two lyso‐PC (LPC) lipids as peaks corresponding to LPC (19:0) and LPC (24:0) were consistently identified in the Sb‐S strain but were absent in the Sb‐R strain. The Sb‐R WT strain had significantly higher levels of eight PCs. All of the PCs upregulated in the Sb‐R strain were unsaturated, with five or more c‐c double bonds in the tail groups.

**Table 2:** Summary of the metabolic and lipidomic differences observed between Sb‐S and Sb‐R strains during the selection of MIL‐resistance.

Standing variation at outset	Metabolites^a^	LPC^b^	PC^b^
WT Sb‐S vs WT Sb‐R differences	14	3	18
Effect of MIL on above differences	N/A	N/A	2 inc. In Sb‐S MIL‐R
New changes during MIL‐R	Metabolites^c^	LPC^d^	PC^e^
Identical in Sb‐S and Sb‐R	8	0	0
Unique to Sb‐S	15	4	11
Unique to Sb‐R	4	5	10

For full data see Tables: ^a^S5, ^b^S6, ^c^S11, ^d^S12, ^e^S13.

### Exposure to MIL produces highly resistant *L. donovani* parasites

**Figure 1:**
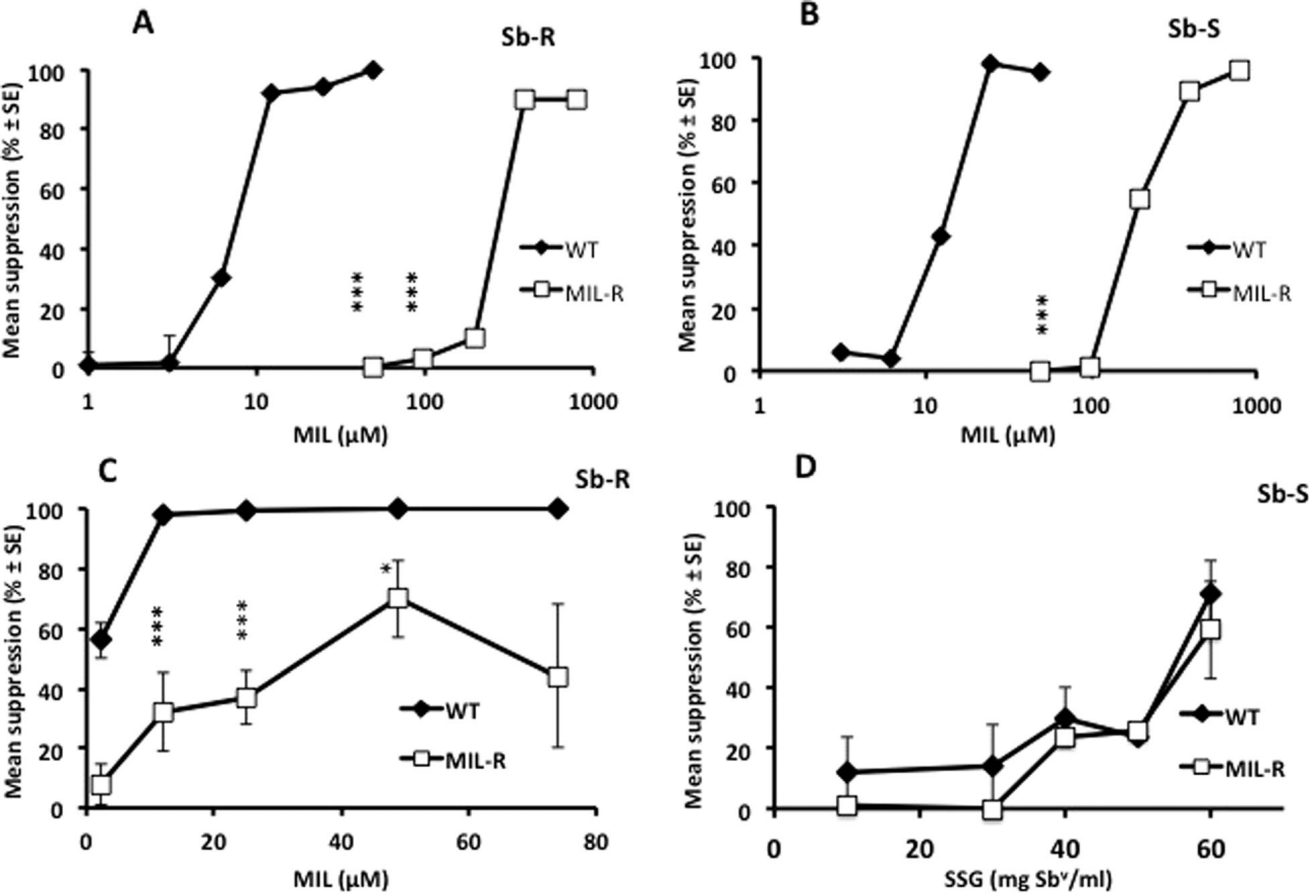
The effect of drug treatment on the survival of promastigotes (A, B) or intracellular amastigotes (C, D) of *L. donovani* parasites treated with MIL (A, B, C) or sodium stibogluconate (SSG, D). Promastigotes of the Sb‐S or Sb‐S MIL‐R clone 8 strain were grown in the presence of medium alone (controls) or different concentrations of MIL (*n* = 6/treatment). Cytotoxicity was assessed by determining the mean suppression of drug treated samples compared to the relevant control (A, B). In amastigotes studies, macrophages were infected with the relevant parasite (C, Sb‐R WT or Sb‐R MIL‐R clone 9, D; Sb‐S WT or Sb‐S MIL‐R clone 8) and then incubated with medium alone (controls) or medium containing MIL (C) or SSG (D, *n* = 4/treatment). After 72 hours, the percentage of cells infected was assessed and the mean suppression in parasite numbers compared to the relevant control. Data is representative of a minimum of two experiments. *P < 0.05, ***P < 0.001 compared with relevant control.

Selection of WT promastigotes that were resistant to the highest concentration of MIL tested (74 µM) took 31 weeks. Both WT strains took similar lengths of time to adapt to the highest concentration of MIL. The susceptibility of the MIL‐R parasites was tested against the intracellular amastigote stage using infected macrophages throughout the experiment to determine if MIL‐R selection at the promastigote stage was conveyed to the intracellular amastigote stage. For both Sb‐S and Sb‐R strains, the IC_50_ values of amastigotes did not increase compared with the corresponding WT until promastigotes tolerated 12.2 µM MIL (Table 3). The fully resistant promastigotes (74 µM) were also clearly resistant at the amastigote stage, with an IC_50_ of 44 µM (15 times the value of WT) and 35 µM (13 times the value of WT) for the Sb‐S MIL‐R and Sb‐R strain MIL‐R, respectively, (Table 3). Increasing the drug concentration to select MIL‐R promastigotes from 49.2 to 74 µM did not result in a corresponding increase in the IC_50_ at the amastigote stage (Table 3). The fact that Sb‐S MIL‐R and Sb‐R MIL‐R parental promastigotes had IC_50_ values that were higher than the maximum MIL concentration used to induce resistance was not unexpected, as all two MIL‐R clones were grown in the medium containing 74 µM MIL. An example of the data obtained is shown in Fig. 1A and B (promastigotes of Sb‐R and Sb‐S strains) and Fig. 1C (amastigotes of Sb‐R strain).

**Table 3:** The effect of MIL selection on the MIL IC_50_ values of WT, MIL adapted parent and clones derived from the MIL‐R parent for Sb‐S and Sb‐R *L. donovani* strains.

Parasite	Mean IC_50_ value (µM ± SE)	N	MIL‐R/WT IC_50_ ratio
**Promastigotes**			
WT Sb‐S	13 ± 0.2	2	
Sb‐S MIL‐R 74 µM parent	371 ± 15	2	28.1
Sb‐S MIL‐R 74 µM clone 2	165 ± 37	2	12.5
Sb‐S MIL‐R 74 µM clone 3	164 ± 1	2	12.4
Sb‐S MIL‐R 74 µM clone 6	185 ± 19	2	14.0
**Sb‐S** MIL‐R **74** μ**M clone 8**	254	1	19.2
WT Sb‐R	6.2	1	
Sb‐R MIL‐R 74 µM parent	357 ± 3	2	57.9
Sb‐R MIL‐R 74 µM clone 3	282 ± 1	1	45.5
Sb‐R MIL‐R 74 µM clone 6	281 ± 1	1	45.8
**Sb‐R MIL‐R 74** μ**M clone 9**	294 ± 6	2	47.4
**Intracellular amastigote**			
WT Sb‐S	3 ± 1	6	
Sb‐S MIL‐R 3 µM	1	1	4.7
Sb‐S MIL‐R 6 µM	2	1	16.1
Sb‐S MIL‐R 12.2 µM	14	1	15.1
Sb‐S MIL‐R 49.2 µM	47	1	15.3
Sb‐S MIL‐R 74 µM parent	44 ± 13	2	
Sb‐S MIL‐R 74 µM clone 8	46 ± 18	2	
WT Sb‐R	3 ± 1	6	
Sb‐R MIL‐R 3 µM	0.2	1	3.3
Sb‐R MIL‐R 6 µM	2	1	12.7
Sb‐R MIL‐R 12.2 µM	9	2	13.4
Sb‐R MIL‐R 49.2 µM	33	1	17.6
Sb‐R MIL‐R 74 µM parent	35 ± 8	2	13.5
Sb‐R MIL‐R 74 µM clone 9	53 ± 1	2	
Sb‐R MIL‐R 74 µM clone 9 no drug	35 ± 1	2	

Clones in bold selected for detailed metabolomic/lipidomic analysis.

The Sb‐S MIL‐R and Sb‐R MIL‐R promastigotes adapted to 74 µM were then cloned to determine if the clones had the same MIL susceptibility as the MIL‐R parent line. These clones had MIL IC_50_ values that were lower than the corresponding MIL‐R parent. This indicated that selection resulted in a mixed population of parasites with different susceptibilities to MIL (Table 2). The clones with the highest resistance to MIL for both strains (clone 8 for the Sb‐S MIL‐R strain, clone 9 for the Sb‐R MIL‐R strain) were then tested for their susceptibility to MIL as intramacrophage amastigotes using *in vitro* infected macrophages. The IC_50_ value of the Sb‐S MIL‐R parent and its clone were similar, and removal of MIL pressure for 2 weeks of culture did not alter the MIL susceptibility of the MIL Sb‐S MIL‐R cloned parasite. In contrast, the cloned Sb‐R MIL‐R parasite had a slightly higher IC_50_ compared to its parent, and culturing the cloned parasite in the absence of MIL resulted in a drop in MIL susceptibility of the cloned parasite to the Sb‐R MIL‐R parent range. Selection of MIL‐R did not impact on Sb susceptibility as the Sb‐R MIL‐R clone 9 gave a similar dose‐response to SSG treatment as the WT Sb‐R parasite (Fig. 1D).

### Genomic changes during MIL‐R selection

The type of strain‐specfic and common genetic changes that occurred after MIL adaption of the two strains are summarized in Table 1.

#### Mutated loci common to both strains

**Figure 2:**
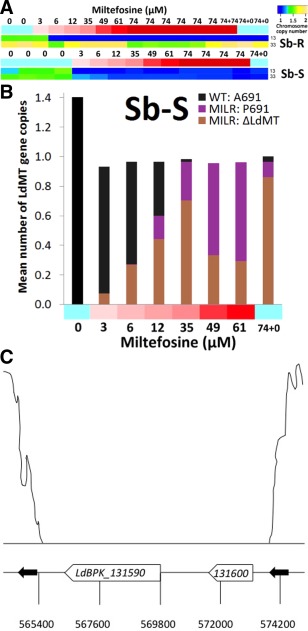
The genetic mechanisms responsible for reducing the amount of LdMT transporter protein in parasites. A. Somy changes observed for Sb‐R WT (top) and Sb‐S WT (bottom) during MIL exposure (x‐axis) ranging from 0 (blue) to 74 µM (shades of red). The WT (0 uM) and fully resistant stages (74 µM) were completed with one and five replicates for Sb‐R, (respectively), and with three replicates for Sb‐S. For Sb‐R, a resistant sample was passaged with (74 + 74) and without the drug (74 + 0, with one replicate). For Sb‐S, a resistant sample was passaged without the drug too (74 + 0). The blue‐green‐beige shading indicates the ploidy state: 1 is disomic, 1.5 is trisomic, and 2 is tetrasomic. Chromosome 13 was downregulated in both strains, Chr33 was only in the Sb‐S one. B. The routes to reducing LdMT dosage for Sb‐S WT during MIL exposure (x‐axis) ranging from 0 to 74 µM: an initial decrease in dose though aneuploidy at 3 µM, a deletion also at 3 µM, and then A691P at 6 µM. The y‐axis indicates the alleles’ copy number within the Sb‐S population assuming one LdMT copy represents the expected disomic state. Black indicates the WT state (A691). At 3 µM MIL, the chromosome copy number decreased from 2.80 to 0.93, and a deletion occurred encompassing LdMT that continued to increase in frequency until 35 µM. At 12 µM MIL, P691 occurred and rose in level until the final step at 61 µM. C. Localisation of the LdMT deletion in the Sb‐R MIL‐R strain; upper part, sequencing read depth over the region; lower part, physical map of the LdMT locus: coordinates were given in a draft reference based on Pacbio sequencing, as this region contains gaps in the current reference and was not properly assembled. The two black arrows indicate the localization of the two 444 bp direct repeats at the deletion boundaries.

Upon selection of MIL‐R, the most significant change shared between the Sb‐S and Sb‐R was of the LdMT locus on chr13 (LdBPK_131590, Supporting Information Tables S7‐S8). In addition to a decrease in the copy number of chr13 (Table 1, Fig. 2A and B), there were three different mutations at this gene: one in the Sb‐R and two in the Sb‐S. The Sb‐R one (E197D) was observed at 35 µM MIL and was present in all cells by that stage, despite only emerging at 12.2 µM. Surprisingly, two independent LdMT variants were discovered in the Sb‐S MIL‐R line: a deletion (ΔLdMT) and a SNP (A691P); Figure 2B shows the evolution of these variants during the selection process. At 3 µM, the 8.86 kb deletion of bases 621,000 to 629,860 encompassing two genes (LdBPK_131590, the LdMT locus at positions 622,408‐625,701 and LdBPK_131600, at positions 627,613‐629,076) was found and it gradually increased in frequency within the population from 7% (3 µM MIL) to 27% (6 µM) to 44% (12.2 µM) to 71% (35 µM). A691P was first observed in the Sb‐S parasite cell population at 12.2 µM MIL with a frequency of 16%, indicating at this stage 36% of the Sb‐S population still survived with the WT LdMT allele (apart from a reduced chr13 copy number) assuming 10 generations/step. By 35 µM, only 2% of the Sb‐S population did not have a mutant LdMT, indicating that a WT LdMT was not viable at this level of toxicity. The fall in population frequency of ΔLdMT from 71% (35 µM) to 33% (49 µM) and 29% (61 µM) was paralleled by a corresponding jump in LdMT‐A691P (62% at 49 µM and 67% at 61 µM respectively). This suggested that A691P was more advantageous at higher concentrations of MIL than the deletion, perhaps due to deleting an unannotated adjacent gene (LdBPK_131600, at 627,613–629,076) or because some unknown function of LdMT may be preserved by the A691P substitution. Subsequent passaging in the absence of drug pressure increased the frequency of the deletion (ΔLdMT, 0.86) and the sensitive allele (LdMT‐A691; 0.04) relative to the resistant LdMT‐P691 (0.10). Sequence alignments showed that the ΔLdMT region adjoining LdBPK_131590 and LdBPK_131600 was flanked by two 444‐bp direct repeats (Fig. 2C).

Changes in mutation frequencies over time during the selection process were used to infer the relative importance of the different MIL‐R mutations using a mathematical model. The evolutionary history of mutations already implicated in MIL‐R indicated that mutations at the LdMT gene were beneficial with a selective advantage of 17% for the deletion and 26% for the A691P allele in Sb‐S. No significant deviations from a neutral model were observed for the other MIL‐R mutations. Our model predicted that the LdMT deletion was pre‐existing in the Sb‐S cell population at a frequency of close to 1% (Supporting Information Fig. S1). By contrast, A691P was inferred to have a frequency < 0.02% in the initial population, suggesting that this could be a *de novo* mutation. Changes in copy number also appear to drive adaptation to MIL (Supporting Information Table S9).

The deletion of LdMT was verified by PCR using genomic DNA prepared from Sb‐S MIL‐R clone 8 and Sb‐R MIL‐R clone 9 promastigotes, and a PCR product was only detected with the latter (data not shown). LdMT expression was verified in WT Sb‐R and Sb‐R MIL‐R promastigotes by quantitative RT‐PCR. LdMT transcripts were present in the WT Sb‐R but not in the Sb‐R MIL‐R clone 9, while both samples showed alpha tubulin transcripts (Ct values for WT Sb‐R, Sb‐R MIL‐R and negative controls, respectively; (i) for alpha‐tubulin: 24.07 ± 0.28; 27.19 ± 0.84; 35.01 ± 0.94 and (ii) for LdMT: 27.37 ± 0.36; > 35. We verified in the Illumina alignments if SNPs were present in the UTRs flanking LdBPK_131590 and did not find any: the next SNP was a synonymous C/A transition at position 575518 of gene LdBPK_131610 (position corresponds to new draft reference genome after PacBio sequencing, unpublished data; it corresponds to position 631021 in the current reference genome).

#### MIL‐selected changes specific to different backgrounds

All the genomic differences observed at the onset of the study between the WT Sb‐S and Sb‐R strains remained upon MIL‐R selection, except the somy of 5 chromosomes (2, 8, 14, 33 and 35, summarized in Table 1). For each sample, a series of specific variants appeared during the selection (Supporting Information Text S1 and S2). When comparing the Sb‐S MIL‐R strain to its WT counterpart, somy was altered in 4 chromosomes (increase of Chr6, 14 and 23 and decrease of Chr33, Supporting Information Table S2), there were 17 heterozygous SNPs (Supporting Information Table S7) and 10 CNVs (Supporting Information Table S10) – the sole homozygous SNP was A691P. For the Sb‐R MIL‐R versus WT comparison, somy was also altered for 4 chromosomes (decrease in Chr2 and 8, increase in Chr9 and 35, Supporting Information Table S2), there were 6 homozygous SNPs (including E197D), 7 heterozygous ones (Supporting Information Table S8), and 10 CNVs (Supporting Information Table S10). Interestingly, all CNVs observed in the Sb‐S MIL‐R strain corresponded to single CDS, while 4 of the CNVs of the Sb‐R MIL‐R strain corresponded to large fragments of chromosomes (amplification of 49,735 bp in Chr27, spanning a whole transcription unit; deletion of 116,778 bp in Chr 31; two deletions of 307,617 bp and 674,372 bp, respectively, in Chr35) containing a total of 298 CDS (Tables 1 and Supporting Information Table S10).

### MIL transporter mutations are likely to prevent phospholipid transport

**Figure 3:**
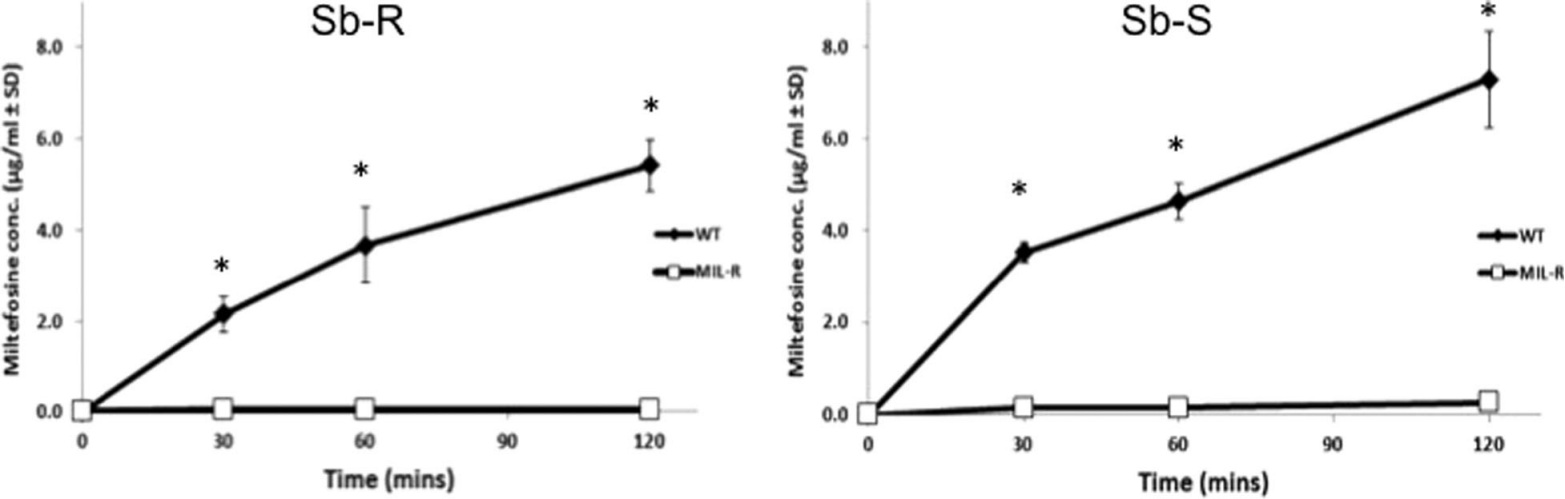
A comparison of MIL uptake in MIL‐R (Sb‐S MIL‐R clone 8, Sb‐R MIL‐R clone 9) and their corresponding WT strains (Sb‐S and Sb‐R) of *L. donovani*. Promastigotes were cultured in HOMEM medium with and without 7 µM MIL for 0‐120 min. Samples were quenched and lipid extraction carried out using promastigotes samples (4 × 10^7^, *n* = 4/treatment). The amount of MIL present (µg/ml) was determined using a calibration curve prepared using MIL standards.

A variety of mechanisms could be responsible for the higher resistance of MIL‐adapted parasites compared with the corresponding WT. One is reduced drug uptake by the MIL adapted parasites compared to the WT. To explore this, we initially monitored MIL levels in Sb‐R WT and Sb‐R MIL promastigotes exposed to MIL. We found that it was only possible to determine drug levels up to two hours post‐treatment as WT parasites began to show signs of cell death after two hours, resulting in smaller number of parasites being present. We then compared MIL levels in drug exposed Sb‐S WT and Sb‐R WT promastigotes and their MIL resistant parasites. MIL concentrations were significantly lower in the MIL‐R promastigotes compared to the corresponding WT parasites (*P* < 0.05) at all times points, indicating that MIL uptake was minimal or even non‐existent in MIL‐R parasites (Fig. 3).

### Metabolomic changes during MIL‐R selection

Twenty‐three metabolites were significantly altered in the Sb‐S MIL‐R clone compared with the Sb‐S WT parent and in all cases adaptation to MIL was associated with an up regulation in the metabolites (*P* < 0.05, Tables 2 and Supporting Information S11). In contrast only 12 metabolites were significantly different in Sb‐R MIL‐R clone compared with the Sb‐R WT, with 9 being upregulated and 3 being down regulated compared to the WT (Supporting Information Table S11). Inspection of the metabolites altered in the Sb‐S and Sb‐R strains (Supporting Information Table S5) and their corresponding MIL adapted strains (Supporting Information Table S11) indicates that differences in the metabolites was not simply due to existing differences in the metabolites present in the WT strains.

**Figure 4:**
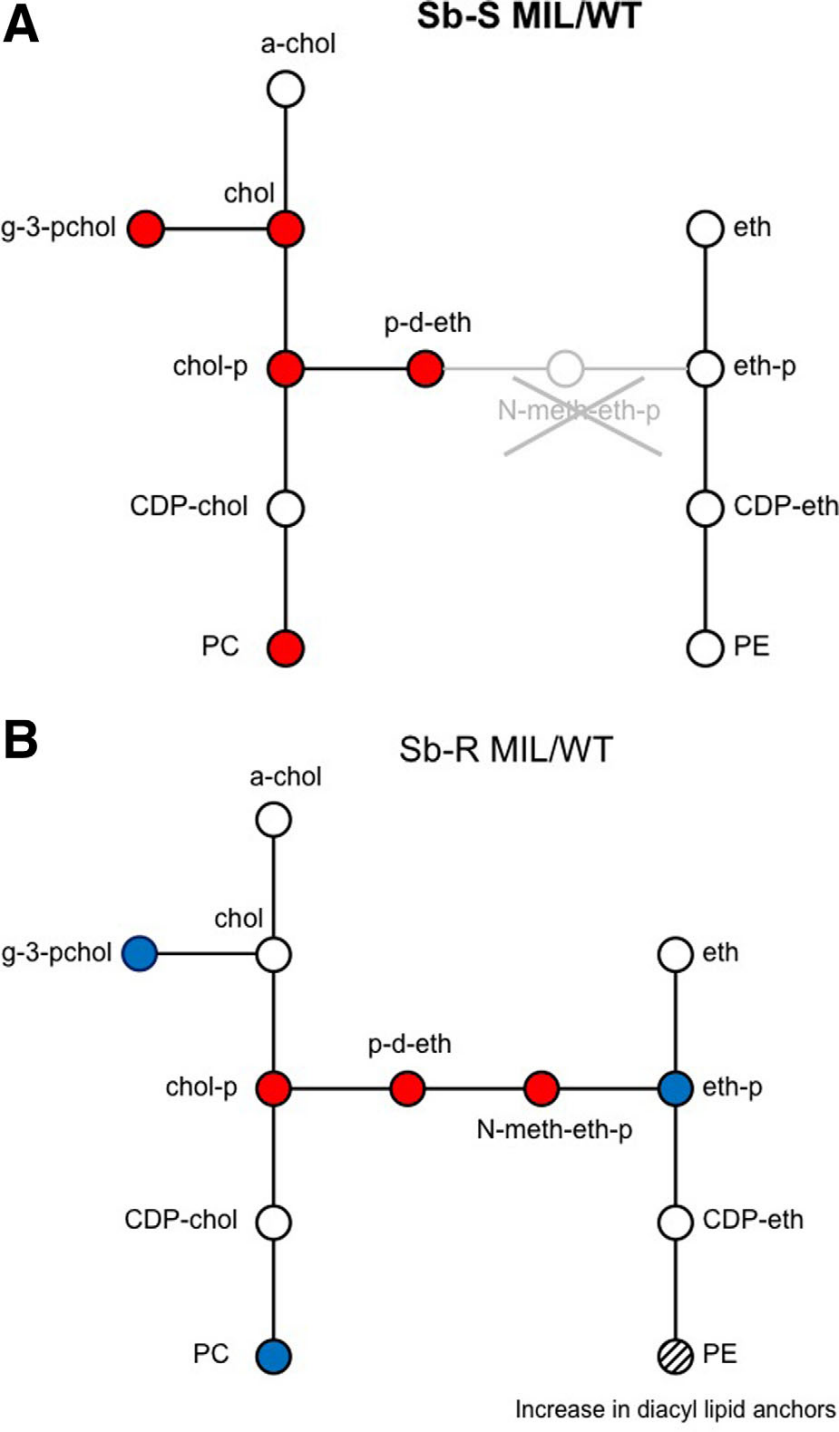
Diagrammatic representation of the Kennedy pathway metabolites altered in the MIL‐R parasites of Sb‐S (A) and Sb‐R (B) compared with their corresponding WT. A blue circle denotes a significant down regulation (*P* < 0.05) of the metabolite in the MIL‐R strain compared to its WT. A red circle denotes a significant up regulation (*P* < 0.05) in the MIL‐R compared to WT. Key: a‐chol, acytelcholine; g‐3‐pchol, glycero‐3‐phosphocholine; chol, choline; CDP‐choline, cytidine diphosphate‐choline; PC, phosphatidylcholine; p‐d‐eth, phosphodimethylethanolamine; N‐meth‐eth‐p, N‐methylethanolamine phosphate; eth, ethanolamine; eth‐p, ethanolamine phosphate; CDP‐eth, cytidine diphosphate‐ethanolamine; PE, phosphodimethyethanolamine.

Nine metabolites involved in lipid metabolism were found to be the affected by selection of MIL resistance between the two strains. Six of these are involved in the Kennedy pathway (Fig. 4), the primary metabolic pathway for the synthesis of PC and phosphatidylethanolamines (PE). Only three of the significantly altered metabolites were common to both MIL‐R clones, that is, choline‐phosphate, phosphodimethylethanolamine (both involved in the Kennedy pathway) and stearoylglycerone phosphate, and they were all upregulated.

### Lipidomic changes during the MIL‐R selection

Selection of MIL‐R was associated with significant differences in lipid content compared to the corresponding WT parent but these were restricted to PC and LPC lipids (summarized in Table 2), and alterations in the composition of PE lipids (data not shown). Four LPCs were significantly upregulated in the Sb‐S MIL‐R strain compared to its WT. Three LPCs were upregulated in the Sb‐R MIL‐R strain compared to its WT, and two were down regulated (Supporting Information Table S13). Two of these altered LPCs were common to both MIL‐R strains [LPC(16:00) and LPC(18:0)], and in both cases the corresponding LPC was upregulated compared to the respective WT.

Eleven PCs were found in significantly higher amounts between the Sb‐S MIL‐R strain and its WT, with six having a ratio of > 2.0. In sharp contrast, all 10 PC were downregulated in the Sb‐R MIL‐R strain compared with its WT (Table S13). None of the altered PCs were common to the two MIL‐R strains (Table S13). Only five PCs altered in the MIL‐R strains were significantly different between the two WT strains (compare Supporting Information Tables S6 and S13). Three PCs were significantly higher in the WT Sb‐S strain compared to the WT Sb‐R strain [PC(32:0), PC(33:0), PC(35:0)] and MIL‐R selection further increased this difference. All of the PCs found to differ between Sb‐S MIL‐R and WT were downregulated, long chain PCs and highly unsaturated (at least 39 carbons in the side chains and 4 to 10 carbon double bonds). In contrast, all PCs significantly different in Sb‐R MIL‐R were upregulated and were either saturated or had only a small number of carbon double bonds (maximum of 4).

Phosphodimethylethanolamines (PEs), diacyl‐glycerols (DAGs), triacyl‐glycerols (TAGs), sphingolipids (SLs) and other minor lipid classes were analyzed for alterations occuring as a result of MIL‐R. Of these, only SLs of Sb‐R MIL‐R were found to differ significantly compared to the corresponding WT. Of the sphingolipids identified in the Sb‐R WT and MIL‐R lines, 15 were consistently downregulated in the Sb‐R MIL‐R compared to Sb‐R WT (*P* < 0.05, Supporting Information Table S14). Of these, eight were different species of sphingomyelins and five were ceramides. Similar levels of these lipids were present in Sb‐S and Sb‐R WTs, indicating that the significant changes in these lipids were specific to the Sb‐R MIL‐R.

## Discussion

We performed a stepwise selection of MIL resistance in WT Sb‐S (BPK282/0cl4) and Sb‐R (BPK275/0cl18) strains derived from recent clinical isolates from Nepal. In the study, amastigote and promastigote MIL sensitivities together with lipidomic, metabolomic and genomic changes possibly associated with MIL‐resistance (MIL‐R) were assessed to explore a possible impact of Sb susceptibility background on the development of MIL‐R. This study represents the first time such a comprehensive biochemical and genomic screening has been performed, and it allowed molecular differences associated with adaptation to MIL to be linked to mutational changes and changes in lipid and metabolite profile within the two strains. In our study we focused our molecular analyses on the promastigote stage rather than the clinically relevant amastigotes, for several reasons. It was impossible so far to select MIL‐R in amastigotes of *L. donovani* (Hendrickx *et al*., 2014); in contrast, in promastigotes, it is rather easy and as demonstrated here, the MIL‐R phenotype is preserved in derived amastigotes. In addition, it is difficult to extract host‐free amastigotes from *in vitro* or *in vivo* infected macrophages and the purification and extraction processes may lead to changes in lipid or metabolite profiles compared to what occurs *in situ*. Results from untargeted studies such as this study using promastigotes can be used in the future to guide targeted studies in amastigotes.

During the selection experiment, we generated parental lines with 28 to 57 times decreased susceptibility to MIL as promastigotes, while decrease was around 13 to 15 times in the amastigote stages. A significant increase in the IC_50_ value for the intramacrophage amastigote stage was not apparent until parasites adapted to survive in 12.2 µM MIL. Studies on the pharmacokinetics of MIL indicate that blood levels above this concentration should be achieved, for example treatment with MIL at 100 mg/kg for 28 days resulted in maximal median concentration of 70 µg/ml (172 µM) on day 23 (Dorlo *et al*., 2012). However blood levels may not be indicative of MIL levels within the macrophage and more recent studies have indicated that MIL treatment failures are due to limited drug exposure in patients (Dorlo *et al*., 2014) rather than MIL‐R in the parasite population. We observed considerable cytotoxicity in macrophages treated with 148 µM MIL, indicating that this concentration would be very cytotoxic if it is maintained *in vivo* after dosing. Therefore intracellular levels are likely to be much lower than 172 µM. Thus, intracellular parasites within the parasitophorous vacuole of infected macrophages may have limited exposure to high concentrations of MIL, and this may explain why it has proved difficult to show increased IC_50_ values in parasites recovered from patients that relapse after MIL treatment (Rijal *et al*., 2013).

Clones derived from the parental MIL‐R lines were also resistant to the drug, albeit at variable degrees, hereby supporting the polyclonal nature of a resistant population with individual cells with varying susceptibilities (Coelho *et al.*, 2012). Our results also demonstrated that the main mechanism responsible for the resistance of our lines was a low accumulation of the drug in MIL‐R parasites. Previous studies showed that this phenomenon can be associated with lower inflow of higher efflux, through alterations of the P‐type ATPase LdMT and/or its beta sub‐unit, LdRos3 (Pérez‐Victoria *et al.*, 2003; 2006) or over‐expression of ABC‐transporters (Castanys‐Muñoz *et al.*, 2008). From a genomic point of view, our results converged on alterations of LdMT in both Sb‐S and Sb‐R strains, and no other miltefosine‐driven changes were detected. Different types of mutations were observed, including non‐synonymous SNPs at different positions of LdMT, complete deletion of the gene, and changes in the copy number of chromosome 13 containing LdMT. Interestingly, the first genomic adaptation during selection was the early (< 12.2 µM MIL) shift of chromosome 13 from trisomy to disomy in both Sb‐S and Sb‐R. Aneuploidy is a major feature of *L. donovani* genetic variation (Downing *et al*., 2011; Mannaert *et al*., 2012) and the ability to alter the chromosome copy number may be a simple mechanism to respond quickly to selection pressures, perhaps acting as transitional intermediate fitness state until more beneficial mutations have occurred (Covert *et al*., 2013).

Loss‐of‐function mutations are common in drug resistant parasites (Ashley *et al*., 2014) and this loss of gene function can offer a path to rapid adaptation to environmental stresses (Khan *et al*., 2011). Deletion of LdMT was observed in the Sb‐S strain only with a changing frequency during the selection process relative to alternative SNP alleles within the population of cells. LdMT was deleted together with a gene encoding a hypothetical protein (LdBPK_131600) and deep analysis of the sequence revealed that the locus containing the two genes was flanked by two 444‐bp direct repeats. Such repeats are abundant in the *Leishmania* genome and by homologous recombination they can lead to amplification or deletion of the corresponding loci (Ubeda *et al*., 2014). Mathematical modeling showed a selective advantage for the deletion in the Sb‐S strain, but the advantage was higher for the A691P SNP. With respect to SNPs, there is no evident pattern in the LdMT mutations’ position on the protein internal or external portion, effect on protein hydrophobicity, proximity to transmembrane (TM) domains (Supporting Information Tables S15–17), or local domain type (Text S3). Last but not least, we did not detect transcripts of LdMT in the Sb‐R MIL‐R strain. This type of phenomenon is generally associated with mutations in the regulatory regions flanking the gene of interest and in present case, the only SNP was within the CDS itself (E197D) and the closest one was a C/A transition in the second next gene (LdBPK_131610). Such a result is rather unexpected, but is similar to studies in trypanosomes where a loss of heterozygocity at the Tb*AT1* locus accompanied loss of expression (Stewart *et al*., 2010).

From a metabolomic point of view, a few metabolites showed similar changes in both MIL‐R strains. Three of them were amino acids, class of metabolites previously reported to be altered in different types of drug‐resistant *L. donovani* lines in which they were associated with protection against oxidative stress (Berg *et al*., 2013, 2015). One of which was proline, which is also associated with protection against oxidative stress (Inbar *et al*., 2013). Another class of metabolites deserves particular attention, that is, those involved in lipid metabolism. More specifically, the Kennedy pathway, the primary metabolic pathway for the synthesis of PCs and PEs in *L. donovani* (Kennedy and Weiss, 1956), appeared to be significantly modulated upon resistance to MIL. While different metabolites of the Kennedy pathway were found to be modulated in both MIL‐R strains, two of them were common, namely choline‐phosphate and phosphodimethylethanolamine. Consistent with this finding, the lipid content of MIL‐R parasites was altered compared to their WT, with PCs and LPCs being mostly affected. Four LPCs and 11 PCs were upregulated in Sb‐S MIL‐R compared to its WT, and 8 of the PCs had between 1 and 4 double bonds in the fatty acid tails. In contrast, most lipids were downregulated in the Sb‐R MIL‐R compared with its WT, their PCs were polyunsaturated and had relatively long chain tails. This indicated that the antimony susceptibility background of the strains did have an impact on the adaptation shown by the parasites. Previous studies have highlighted an altered lipid metabolism in MIL‐R parasites and MIL treatment has been associated with a decrease in PC content in tandem with an increase in PE content. (Rakotomanga *et al*., 2007; Imbert *et al*., 2012). This is somewhat correlated with our Sb‐R MIL‐R clone in so far as a decrease in PCs are observed. However a significant increase in PC content was the most notable change in the lipid pool for the Sb‐S MIL‐R. This inconsistency may reflect the difference in the genetic background of the parasites. Rakotomanga and Imbert's studies used strain LV9 [MHOM/LV9/HU3] from Ethiopia whereas clinical isolates from Nepalese VL patients were used in this study. The compositions of lipids in the parasite membrane are known to have a significant effect on the membrane fluidity (Berg *et al*., 2013, 2015). Increasing the chain length or saturation of fatty acid tails can also effect membrane fluidity and increase resistance to oxidative stress, making the parasites even better at tolerating exposure with reactive oxygen species within the host macrophage (Zhang and Beverly, 2010). Sphingolipid metabolism was also significantly reduced in Sb‐R MIL‐R. Given that SLs are a major source of ethanolamine phosphate in the Kennedy pathway, it would be logical to see a knock on effect in the PE content. While the total PE content of Sb‐R MIL‐R appeared unaffected, the composition of the lipid anchors altered with a significant increase in PEs with diacyl lipid anchors. PEs can be synthesized via headgroup exchange with PCs, and the significant decrease of diacyl‐PCs in Sb‐R MIL‐R compared to Sb‐R WT could be due to conversion of these PCs to PEs, which would explain the reduction in SL metabolism in Sb‐R MIL‐R.

This study identified a number of genetic, lipidomic and metabolomic changes during MIL adaptation in two recent clinical isolates with differing prior sensitivities to antimonials. Both isolate types adapted at similar rates to MIL and could tolerate it at comparable levels. This study highlighted that both shared a two‐tiered genetic response, first altering chromosome copy number and second mutating the LdMT gene when more MIL was added. These changes were paralleled by metabolic and lipid ones focused on the Kennedy pathway. More metabolite changes occurred in the Sb‐S strain compared to the Sb‐R one, suggesting that Sb‐R was associated with a faster development of MIL‐R. However, the picture was different from a genomic point of view, specifically at CNV level (298 in the Sb‐R MIL‐R strain vs 8 only in the Sb‐S MIL‐R one, Table 1). We found initial evidence that antimonial resistance does not provide immediate faster adaptations to high doses of MIL. Future studies should address the lack of knowledge of the biochemical pathways of *Leishmania* and their regulation to improve understanding of the molecular mechanisms responsible for MIL resistance. Our study focused on two isolates but we have identified key metabolites, lipids and genetic changes for future studies using more strains. In addition supplementing the medium with radiolabelled precursors (Millerioux *et al*., 2013) and subsequent identification of metabolite/lipid products in the Kennedy pathway, would allow more detailed analysis of the impact of MIL‐R on the parasite's biochemistry.

## Experimental procedures

### Materials

Miltefosine was kindly provided by WHO‐TDR. Resazurin, Giemsa stain, were purchased from Sigma‐Aldrich (Gillingham, UK). HOMEM medium (custom made by Invitrogen, Paisley UK), RPMI‐1640, penicillin/streptomycin, glycine and foetal calf serum were obtained from Invitrogen, Paisley, UK. All organic solvents (acetonitrile, isopropanol, methanol, chloroform) were HPLC grade (Fisher Scientific, Loughborough, UK). Ammonium formate was HPLC grade (17843, Sigma‐Aldrich Company, Gillingham, UK) and mass spectroscopy vials (KVP6112) fitted with a low‐volume insert (INSF‐01) were purchased from Kinesis(St Neots, UK). Isolation of DNA and RNA was performed using DNeasy Blood & Tissue kit or RNeasy Mini kit, respectively, purchased from Qiagen, (Crawley, UK) and cDNA synthesis was performed using AffinityScript Multiple Temperature Reverse Transcriptase (Stratagene/Agilent Technologies, Wokingham, UK). Random Primers (Bioline Reagents, London, UK). MyTaq DNA polymerase reagents (Bioline Reagents) were used for all standard PCR reactions. Real Time PCR reactions were performed using SYBRgreen Master Mix (Abgene/Thermo Scientific, Loughborough, UK).

### Animals and parasites

Age matched inbred BALB/c female mice (20–25 g) in‐house bred were used in studies at Strathclyde University. Animal studies were carried out with local ethical approval and had UK Home Office approval. We used two clones derived from Nepalese clinical isolates and previously tested for their SSG susceptibility (Rijal *et al*., 2007): MHOM/NP/03/BPK282/0cl4 (designated Sb‐S, based on its sensitivity to Sb^V^ and Sb^III^) and MHOM/NP/03/BPK275/0cl18 (designated Sb‐R, based on its relative insensitivity to Sb^V^ and Sb^III^). Both parasites are representative of main populations circulating in the ISC.

### Selection of MIL resistant (MIL‐R) clones

Selection was done on the promastigote stage: *L. donovani* Sb‐S and Sb‐R strains were adapted to grow in increasing concentrations in a step‐wise manner with MIL (3, 6, 12.2, 35, 49.2, 61 µM and 74 µM) until all lines grew at similar rates as wild‐type parasites in HOMEM medium supplemented with 20% foetal calf serum. Promastigote resistance to MIL was stopped at 74 µM because of toxicity for macrophages at higher concentrations. Clones derived from the MIL‐R lines were isolated using a micro‐drop method (Van Meirvenne *et al*., 1975), and expanded in Tobie's medium and then passaged using HOMEM medium supplemented with 20% (v/v) foetal calf serum. A stock solution of aqueous MIL (3 mg/ml, freshly prepared every 3 months) was stored at 4°C was used to prepare drug selection medium.

### Lipidomic/metabolomic studies

Extraction of lipids or metabolites was carried out on day 4 of culture. Prior to extraction all of the biological replicate cultures were quenched to 0°C in an ethanol‐dry ice bath to halt metabolism. For each strain, four aliquots, from four separate cultures, containing 4 × 10^7^ promastigotes, were pipetted into pre‐chilled eppendorf tubes which were kept at 0°C throughout the extraction procedure. The aliquots were centrifuged for 3 min at 2,700 x g and the supernatant was removed as spent medium for later analysis. The promastigotes were then washed by re‐suspending them in 1 ml of PBS, pre‐chilled to 0°C. They were then centrifuged at 2,700 x g for 3 min and the supernatant was removed. This washing procedure was repeated three times. After washing the parasites were resuspended in 200 µL of methanol and chloroform (1:1 v/v for lipid extraction) or chloroform:methanol:water mixture (20:60:20 v/v for metabolite extraction) and shaken in a thermomixer for 1 hour at 1400 rpm, 0^o^C. The tubes were then centrifuged for 2,700 x g for 3 min at 0°C and the resulting supernatant was removed and transferred to a mass spectrometry vial fitted with a low‐volume insert. Samples were stored at −70°C until analyzed.

Lipidomic and metabolomic samples were analyzed using a Dionex Ultimate 3000 HPLC system (Thermo Fisher Scientific Inc., Waltham, USA). A silica gel column (150mm x 3mm x 3µm, HiChrom, Reading, UK) was used for lipidomic analysis and a ZIC‐pHILIC column (150 mm × 2.1 mm × 5µm, Merck, Darmstadt, Germany) was used for metabolomic analysis. The columns were connected to a Thermo Scientific Exactive Mass Spectrometer (Thermo Fisher Scientific Inc., Waltham, USA) running in positive/negative scanning mode. In lipidomic analysis two solvents were used in separation (solvent A: 20% isopropyl alcohol (IPA): 80% acetonitrile; solvent B: 20% IPA: 80% ammonium formate [20 mM]) using gradient elution at a flow of 0.3 ml/min (0–1 min 8% B, 5 min 9% B, 10 min 20% B, 16 min 25% B, 23 min 35% B, 26–40 min 8% B). In metabolomic studies different solvents were used in separation (solvent A: 20mM ammonium carbonate adjusted to pH 9.2 with ammonia; solvent B: 100% acetonitrile). Gradient elution at a flow rate of 0.3 ml/min was used (0 min 80% B, 30 min 20% B, 31 min 8% B, 36 min 8% B, 37 min 80% B, 46 min 80% B). Processing of metabolite and lipid data was performed as detailed in (t'Kindt *et al*., 2010). Briefly, raw data files produced by the Exactive mass spectrometer were converted to mxXML format and centroided using MSConvert (Proteowizard, http://proteowizard.sourceforge.net/downloads.shtml, accessed on 16 November 2014). Individual peaks were picked based on non‐linear retention time alignment, feature detection and feature alignment using the centwave method from XCMS (https://metlin.scripps.edu/xcms/). These peaks were converted to .peakML files using MzMatch.R (http://mzmatch.sourceforge.net/index.php). MzMatch.R was then used for peak extraction, filtering, normalisation, group filtering and gap‐filling of peaks. This produced a single file in ‘.text ‘format containing peak groups with the same mass/charge (m/z) values from the different biological replicates. This file was then converted to a .txt file and m/z values were identified as specific lipids using Xcalibur and an in‐house Excel macro that calculated the total number of carbons and saturated carbon bonds in the fatty acid tails of each lipid class. Metabolites were identified using IDEOM (Creek Barrett, 2014) and 4 standard mixes were used in assays. The software identified metabolites/lipids that were significantly different between treatments. Comparisons between groups were based on the ratio of WT/WT or MIL‐R/WT, using the mean ratio for the, respectively, WT used as comparator.

### Genome sequencing and variant screening

Genomic DNA was sheared into 400–600‐base pair fragments by focused ultrasonication (Covaris Adaptive Focused Acoustics technology, AFA Inc., Woburn, USA) and standard Illumina libraries were prepared. 100 base pair paired end reads were generated on the HiSeq 2000 according to the manufacturer's standard sequencing protocol (Bonner *et al*., 2009) to produce an average of 55.4‐fold read coverage per site with at least 21‐fold median depth for each sample (Supporting Information Table S1). ENA SRA accession numbers for all sequencing libraries are listed in Table S1. The DNA reads were screened for contamination, PCR duplicate were removed, and those with insert sizes < 1,000 bases were mapped using Smalt with exhaustive alignments (version 0.7.2 www.sanger.ac.uk/resources/software/smalt/, Ponstingl and Ning, 2010). Non‐mapping reads, low quality bases, bases in repetitive regions, bases with poor mapping scores, bases in low‐complexity regions and bases with low read coverage were all excluded from analysis. Bases with significant forward‐reverse strand amplification bias were a result of reads mapping mainly to a single DNA strand and though rare were eliminated by determining the rate of variants as a function of this strand bias effect.

We scanned for dose‐dependent, heterozygous and homozygous mutations because these may be haplosufficient, and proteins associated with resistance may form heterodi‐ or multi‐mers (Paape and Aebischer, 2011). Chromosome copy number variation and scans for large CNVs and episomes were performed based on per cell read depth to reflect gene dosage as outlined previously (Downing *et al*., 2011). Using the difference in read‐depth allele frequencies between steps, 53% of valid SNPs were homozygous (a read‐depth frequency > 0.95). This approach detected heterozygous alleles on trisomic and tetrasomic chromosomes where differences in the allele frequencies based on the reads can be more challenging to detect if present on a single chromosome only. Multiple complementary variant‐calling tools were used to discover SNPs and infer genotypes: Samtools pileup v0.1.11 (Li *et al*., 2009), Samtools mpileup v0.1.18, FreeBayes (Garrison and Marth, 2012), GATK (McKenna *et al*., 2010) and Cortex (Iqbal *et al*., 2012). SNPs associated with 74 µM MIL resistance were present in all technical replicates.

Candidate MIL‐R mutations were identified using a deep sequencing approach using promastigote parasites from each selection step after cloning of the parental parasite, that is, after adaption to 3, 6, 12, 35, 49, 61and 74 µM MIL. Four (Sb‐S MIL‐R clone 8) and six (Sb‐R MIL‐R clone 9) replicates were sequenced to verify the mutations present in the MIL‐R parasites. We also determined the effect of removing drug pressure on the genotype of the Sb‐S and Sb‐R MIL‐R cloned parasites, by maintaining them for two weeks without drug pressure (74 + 0 µM MIL). WT parasites with the same passage number were sequenced at the same time as the corresponding MIL‐R parasites to ensure that polymorphisms associated with long‐term passaging were omitted from drug association. Chromosome copy number (somy), local gene copy number variation (CNV) including extra‐chromosomal segments such as episomes, single nucleotide polymorphisms (SNPs) and indels were examined at each experimental stage. Mutations altering the amino acid code are more likely to have a pronounced positive or negative effect on protein function, and changes unique to the MIL‐resistant clones must be either beneficial or neutral (Weinreich *et al*., 2006). Consequently, nonsynonymous mutations distinguishing MIL‐R from WT consistent across each step of the selection processes were more likely to be truly implicated in MIL resistance.

### Statistical inference of the selective benefit of resistance mutations

A likelihood framework was used to evaluate the mean role of selection acting upon genetic variants within the population (Illingworth and Mustonen, 2012), including both mutation frequencies and somy variants. Given a set of observed data *O*, parameters within a variety of models *M* were optimized to maximize the likelihood *L(O*|*M)*. Different models described different patterns of evolution under neutrality, or selection for specific genetic variants. The Bayesian Information Criterion (BIC) was used to distinguish between models of differing complexity, minimising the value:

BIC = −2 log L(O|M) + k log n

where *n* is the number of data points in the model and *k* is the number of model parameters. Observed allele frequencies were modelled using a multinomial likelihood function, while observed changes in chromosomal copy number were modelled using a double Poisson likelihood function. Full details of the model are given in Supporting Information Text S4.

### Prediction of protein mutation amino acid properties, position and functional impact

The biochemical and structural properties of proteins were examined using amino acid hydrophobicity to determine the TM portions of the protein using Bioedit v7.0.9.1, ProtScale (Gasteiger *et al*., 2005) and the Membrane Protein Explorer (MPEx v3.2, Snider *et al*., 2009). Surface accessibility and secondary structure were used to predict whether mutant amino acids were more likely to be internal or external in the protein, and if they were part of alpha‐helix, beta‐sheet or coil structures (NetSurfP, Petersen *et al*., 2009). Protein function impacts were predicted with Polyphen‐2 v2.2.2 (Adzhubei *et al*., 2010), SNAP (Bromberg and Rost, 2007) and SIFT (Kumar *et al*., 2009).

### PCR studies

PCR studies were completed using genomic DNA or cDNA prepared from RNA, both isolated using *L. donovani* promastigotes. Genomic DNA was prepared using the DNeasy Blood & Tissue kit and RNA was isolated using the RNeasy Mini Kit. Complementary DNA was synthesized from 2 µg of RNA, using 1 µl of Random Primers diluted with molecular grade water to give final volume of 15 µl. The mixture was incubated at 65°C for 5 min and samples were then left at room temperature for 10 min before adding 2 µl of 10X Affinity script buffer, 2µl of 100 mM DDT, 0.8µl of 100mM deoxynucleotide triphosphate (dNTP) mix and 1µl of RT enzyme to a final volume of 20 µl. Samples were incubated at 25°C for 10 min, followed by 55°C for 1 hour, and then heated at 70°C for 15 min. PCR studies with primers specific for *L. donovani* LdMT gene (LdMT‐Forward: 5′‐CAAGTGCCTTTCCACCAGAATC‐3′,LdMT‐Reverese: 5′‐CTCACCTTTTTGAACTCCAAC AGG‐3) and alpha‐tubulin gene (AlphaTubulin‐Forward: 5′‐AGCTGTCCGTCGCGGACATCA CGAACTCGGTGTTT‐3′, AlphaTubulin‐Reverse:5′‐CGAACTGAATTGTGCGCTTCGTCT TGATCGTCGCAAT‐3’). Each PCR reaction contained 2 µl of MyTaq DNA Polymerase, 10 µl of 5x Reaction Buffer, 25 of pmol each of forward and reverse primers, 2 µl of DNA and made to a final volume of 30 µl with molecular grade water. PCR conditions were as follows: Denaturation at 94°C for 3 min, 35 cycles of 94°C for 30 sec, annealing 64°C for 45 sec and extension at 72°C for 1 min. Final extension was carried out at 72°C for 10 min. PCR products were separated by gel electrophoresis and the products visualized under UV light and a digital image of the results obtained.

Quantitative RT‐PCR reactions were carried out in a final volume of 12.5 µl, using 6.25µl of SYBR green, 1 µl of template cDNA, 25 of pmol of forward and reverse primers (as above) and molecular grade water. In RT‐PCR reactions the following conditions were used: denaturation at 95°C for 10 min; 40 cycles of the following steps: 95°C for 30 sec, 63°C for 45 sec and extension at 72°C for 1 min; and a final cycle of 95°C for 30 sec and 55°C for 30 sec. All samples were carried out in duplicate. Gene expression was calculated using the ^−ΔΔC^_T_ method (Livak and Schmittgen, 2001) method. The housekeeping gene α‐tubulin was used as a comparison for expression levels.

### Promastigote inhibition studies

The effects of MIL on the growth of promastigotes were tested using a colorimetric‐based assay using resazurin. Parasites were added to the wells of a 96 well tissue culture plate at a concentration of 5 × 10^5^ parasites/well and grown in the presence of medium alone (controls) or medium containing various concentrations of MIL (3–785 μM, *n* = 6/treatment). Plates were incubated for 72 hours at 27°C. and then 20 µl of resazurin solution (0.0125% w/v PBS pH 7.4) was then added to samples and the samples were incubated for a further 18 hours at 27°C. The absorbance of samples at 550–590 nm was determined using Softmax Pro 2.0 software. The effect of drug treatment on cell viability was determined by calculating the percentage suppression in cell growth for drug treated samples compared with the mean control value (*n* = 6/treatment). The mean suppression/treatment was used to determine the IC_50_ for a particular formulation using Grafit^®^ 5 software.

### MIL uptake studies

To quantify the uptake of MIL by WT and MIL‐R parasites, 5 ml cultures of parasites at day 4 of growth were pelleted, resuspended in 5 ml medium containing 7 µM of MIL, and the resulting suspension was transferred to a new tissue culture flask. Flasks were quenched at time 0, 30, 60 and 120 min post exposure to MIL and 4 × 10^7^ parasites were aliquoted into pre‐chilled eppendorfs, sitting on ice. Lipid extraction was then carried out on these samples as described above. A calibration curve was produced by spiking samples of the extraction solvent with MIL standards at 0.24, 0.72, 2.4, 7.2 and 14.4 mM. Linear regression was used to fit a standard curve for the AUC of the MIL peak from the standards (correlation coefficent > 0.97), and this data was used to determine the MIL present in parasites at time of quenching.

### Macrophage studies

Peritoneal macrophages, harvested from mice 72 hours after intraperitoneal injection of starch solution (3% w/v PBS pH 7.4), using the method described by Carter *et al*. (2005). Cells were infected using a host: parasite ratio of 20:1. The effect of treatment on parasite survival was determined as the mean percentage suppression in the percentage of cells infected and the number of parasites/host cell/for each treatment (*n* = 4/treatment) by comparing each experimental value with the relevant mean control value.

### Statistical analysis of data

The effect of drug treatment on cell proliferation in *in vitro* drug studies were analyzed using a Mann Whitney U test for comparing two treatments or a Kruskal Wallis test followed by Dunns ad hoc test for statistical differences between three or more treatments (Statview^®^ v5.0.1). Lipidomic and metabolomic data which compared WT with WT or WT with the corresponding MIL‐R were analyzed using a Mann Whitney U‐test or a Student's t‐test where the *P*‐values were corrected for multiple testing using the Benjamini‐Hochberg method using R. The null hypothesis was not accepted if the adjusted *P* < 0.05.

## Supporting information

Supporting Information

Supporting Information
