# Supplementary material for: In vitro selection of miltefosine resistance in promastigotes of Leishmania donovani from Nepal: genomic and metabolomic characterization
Source: Mol Microbiol. 2016 Feb 9;99(6):1134–48. doi: 10.1111/mmi.13291 (PMC4832254; doi:10.1111/mmi.13291)
Supplement: Supplementary file 1 — Supporting Information [file MMI-99-1134-s001.docx]

Supplementary Data for:

**‘*In vitro* selection of miltefosine resistance on promastigotes of *Leishmania donovani* from Nepal: genomic and metabolomic characterisation.**

**.’**

**”.**

**Page Text**

1 Text S1. Miltefosine resistance in an antimony-sensitive population.

2 Text S2. Miltefosine resistance in an antimony-resistant population.

3 Text S3. Structural and functional effects of miltefosine transporter mutations.

4 Text S4. Details on quantifying selective benefits of resistance mutations.

**Tables**

6 Table S1. Illumina Hiseq DNA read sequence output for each sample.

7 Table S2. Chromosome copy numbers in BPK282/0cl4 (Sb-S) and BPK275/0cl18 (Sb-R), before and after the MIL-R selection (respectively WT and MIL-R).

8 Table S3. SNPs between WT Sb-R (BPK275/0cl18) and WT Sb-S (BPK282/0cl4).

9 Table S4. Genetic variants between WT Sb-R (BPK275/0cl18) and WT Sb-S (BPK282/0cl4), including (A) non-synonymous SNPs, (B) CNVs (expressed per haploid genome) and (C) indels.

12 Table S5. SNPs observed during MIL induction for Sb-S BPK282/0cl4.

13 Table S6. SNPs observed during MIL induction for Sb-R BPK275/0cl18.

14 Table S7. CNV observed upon MIL-R selection of (A) the Sb-S strain and (B) the Sb-R strain.

15 Table S8. Comparison of metabolites present in Sb-S and Sb-R WT *L. donovani* promastigotes.

16 Table S9. The relative concentration of different lipids present in Sb-S and Sb-R WT *L. donovani* promastigotes parasites.

16 Table S10. Likelihoods for alternative selection models acting on mutations

17 Table S11. The relative concentration of different metabolites present in Sb-S and Sb-R WT *L. donovani* promastigotes parasites.

18 Table S12. The relative concentration of different phosphatidylcholines present in WT Sb-S and Sb-S MIL-R *L. donovani* promastigotes parasites.

18 Table S13. The relative concentration of different lysophosphatidylcholines present in WT Sb-R and Sb-R MIL-R *L. donovani* promastigotes parasites.

10 Table S14. The relative concentration of different of sphingolipids in Sb-R MIL-R compared to Sb-R WT *L. donovani* promastigotes parasites.

20 Table S15. Predicted LdMT protein functional impacts by different mutations.

21 Table S16. Predicted LdMT protein surface accessibility.

22 Table S17. Predicted transmembrane segments of the LdMT protein.

**Figures**

23 Fig. S1. Modelled allele frequency changes at the LdMT gene.

24 **References**

**Text S1. Miltefosine resistance in an antimony-sensitive population.**

The mutations in Sb-S line BPK282/0cl4 detailed below are those not preserved consistently at higher MIL concentrations or that were heterozygous. As outlined in the main paper, at 3 µM in the Sb-S population, there was a downregulation of chr13 and chr33 and a deletion of the LdMT gene (ΔLdMT) (Table S4). Additionally, an amastin gene increased in dose (LdBPK_342650, 3.56 to 4.54). In addition to the downregulation of chr13 at 6 µM, the copy number of a tagatose-6-phosphate kinase was reduced from 6 to 5 (LdBPK_252550, EC 2.7.1.144, Pfam PF00294), which was incompletely maintained in parasites adapted to higher doses of MIL. At 6 µM, a heterozygous SNP at 5’ end of a hypothetical gene also was found (LdBPK_271870).

At 12 µM there was an increase in the IC_50_ for the intra-macrophage amastigote stage, a heterozygous SNP at 5’ end of a hypothetical gene (LdBPK_201010) occurred and CNVs upregulated hypothetical genes on chr6 from two to three copies (LdBPK_060130, LdBPK_060700, LdBPK_061140 and LdBPK_061170). At 35 µM, hypothetical gene LdBPK_061300 was upregulated by approximately one copy (from 1.20 to 1.58 at 35 µM to 1.39 at 49 µM). At 49 µM, a heterozygous 5'UTR was found in a protein kinase gene (LdBPK_141140). At 61 µM, a fatty acid elongase (LdBPK_140770, EC 2.3.1.119) that had two copies in 3-49 µM MIL samples had reverted to three, but this may be a WT state. In addition to two heterozygous synonymous SNPs at hypothetical genes (R560 in LdBPK_110430 and T745 in LdBPK_220160), a two heterozygous nonsynonymous SNPs were found that were predicted to impair protein function (PolyPhen-2 v2.2.2) but these all reverted to the WT genotypes at 74 µM (R620W in hypothetical gene LdBPK_120570, and A216D in vacuolar ATP synthase subunit b, LdBPK_282610).

At 74 µM, three protein-level SNPs were found. The first (*552Y) was in a LdBPK_160690 as pre the main text. The second may alter the RNA product of a hypothetical gene (LdBPK_333250) by reducing it by 315 codons from a total of 1458 (S1143*, Figure S10). And the third was A380T in a protein kinase (LdBPK_320820, EC: 2.7.1.37): but this is predicted to have no functional effect (PolyPhen-2 v2.2.2). The five silent SNPs included one synonymous (F2447 in LdBPK_261790) and three 3’ UTR mutations (LdBPK_160820, LdBPK_242120, LdBPK_311550) were observed, all of which were hypothetical genes. A 3’ UTR SNP was found at base +361 in ABCC5 (LdBPK_311300): this gene has been linked to SSG-R (Leprohon et al 2009b). Kinesin K39 (LdBPK_141180) may be involved in intracellular transport (Pfam PF00225) and increased in copy number from 3-5 copies to 5-6.2. A hypothetical gene (LdBPK_261780) was marginally downregulated. In addition, 27 CNV and 8 SNP differences were found: all but one of the CNVs were increased in dose from two to three copies (except where stated), and all SNPs were heterozygous affecting one chromosome only. Maintaining the MIL-R Sb-S population without MIL pressure presented two new SNPs, both at the 5’ end of one gene encoding a Leucine rich repeat protein (LdBPK_030110); the other was a hypothetical gene (LdBPK_364290).

**Text S2. Miltefosine resistance in an antimony-resistant population.**

The polymorphisms in Sb-R line BPK275/0c18 detailed below are those not preserved consistently at higher MIL concentrations or that were heterozygous. At 3 µM, amplification of hypothetical gene (LdBPK_290110) and downregulation of small nucleolar (sno) RNA 4 on chr30 (LdBPK_30snoRNA4) was detected, but these changes were not maintained about 12 µM. Two silent heterozygous SNPs occurred: one at T511 in poly(A) polymerase LdBPK_141260, the 5' end of a hypothetical gene (LdBPK_341810). At 6 µM MIL, chr31 was also downregulated to trisomy but reverted to tetrasomy at 12 µM. At 12 µM MIL, the mutations are detailed in the main text.

At 49 µM there was a mutation resulting in a heterozygous SNP at the 5' end of a gene encoding an EF hand protein (LdBPK_131490) that may be involved in calcium ion binding (Pfam PF00036), and is orthologous to a *L. braziliensis* programmed cell death 6 gene. At 61 µM, the deletion on chr31 also had a gene with a c2 domain (LdBPK_310820) whose orthologs are involved in calcium-dependent phospholipid binding (Davletov and Sudhof 1993). A number of other hypothetical genes with functionally informative domains derived from Pfam were located in this deletion: LdBPK_310630 may act as a signal peptidase; LdBPK_310640 and LdBPK_310780-90 may be involved in the cell cycle; LdBPK_310680 could have methyltransferase activity; and LdBPK_310770 is similar to a chaperone associated with virulence in *E. coli*. And at 74 µM, a CNV at hypothetical gene LdBPK_110010 was upregulated from ~3 copies (at 3-61 µM) to ~4 copies, though it is not clear which is the WT state.

**Text S3. Structural and functional effects of miltefosine transporter mutations.**

The mutations occurring at LdMT in this experiment (E197D, A691P) and in work on other *L. donovani* isolates (L832F in MHOM/ET/67/HU3, Cojean et al 2012) are notable compared to previous work with *L. major* (Coelho et al 2012) because of the absence of stop codons that might have affected RNA expression of the local syntenic genes at LdBPK_131470-LdBPK_131580. However, earlier work on *L. donovani* has identified an extensive range of SNP types (non-sense mutations at 145 and 210; G276V; L366P; F414S; T420N; F430S; A653V; L780P; G824D; L856P; I914T; Perez-Victoria et al 2003, Perez-Victoria et al 2006).

Protein sequence alignment indicated all *L. donovani* and *L. major* amino acids were orthologous (T-Coffee v9.03.r1318, Notredame *et al*., 2000). We examined the biochemical and structural properties of the LdMT protein by using amino acid hydrophobicity to determine the transmembrane (TM) portions of the protein using Bioedit with Kyte and Doolittle (Kyte and Doolittle, 1982) scaled mean values. These were confirmed with ProtScale with a window size of 20 amino acids as well as Membrane Protein Explorer using default settings (Table S13). There were 13 predicted TM helical regions predicted by MPEx, 12 by TMPred (http://embnet.vital-it.ch/software/TMPRED_form.html, Hofmann and Stoffel, 1993) and 8 by TMHMM v2.0 (www.cbs.dtu.dk/services/TMHMM/, Krogh *et al.*, 2001). 7 were common to all three tools (Table S5) and TMPred most closely matched previous TM domain predictions based on the human ATPase ortholog (Perez-Victoria *et al.,* 2003). No closely related orthologous proteins domains exist for LdMT on which to model its 3D structure.

We explored the surface accessibility and secondary structure predictions for each mutant amino acid to assign whether it is likely to be internal or external on the surface of LdMT (Table S6), and if it forms part of an alpha-helix, beta-sheet or coil structure (NetSurfP). No preference for mutations at the protein surface was evident, consistent with the proposal that all these mutations prevent LdMT function, rather than altering its ligand interactions. Further support for this comes from the wide range of amino acid property changes between the WT and variant alleles, and also from the large number of affected secondary structures. Similarly, the mutant amino acids were intracellular, in TM domains and extracellular, and were present at sites towards both N- and C-termini.

Predictions of the impact of different mutations on the functionality of the protein product indicated that most would halt normal function, but that some were ambiguous across the three tools used: Polyphen-2, SNAP and SIFT (Table S7) – mutations E197D, F414S, A691P and L832F. Except for L832F, only SIFT predicts that E197D, F414S and A691P would not impact protein function. These predictions support the proposition that these single amino acid changes prevent normal LdMT function and therefore prevent miltefosine transport into the cell.

**Text S4. Details on quantifying selective benefits of resistance mutations.**

A likelihood model was used to identify the extent of polymorphism and selection acting upon genetic variants in the population. Our approach exploits the availability of time-resolved data from the population. We note that, when considering selection, the changing drug concentration in the experiment may impose a varying selective force on the population; here, our models of constant selection estimate the mean force of selection acting upon a particular allele or copy number variant across the period of the experiment.

Our method was similar to a previous method for the analysis of the force of selection acting upon neutral marker data (Illingworth and Mustonen, 2012). In general, given a population containing variants {*x_i_*}, we assume that each variant *x_i_* evolves deterministically under the selection coefficient *s_i_*; that is, the evolution of the variant frequency between successive discrete times *t_k_* and *t_k+1_* is given by:

In each case, a single selection coefficient is set to equal zero without loss of generality. This model assumes a large population size, such that the role of selection acting upon the population greatly outweighs the effect of genetic drift. For this reason, data was only considered up to the time of treatment of the population with 61 *μM* miltefosine. After this point, the population was bottlenecked to a small size, such that no further analysis using this method was possible.

Models considered had different numbers of variants *x_i_*. For example, in the case of a single chromosomal copy number change, three models were considered:

i) with a single variant, with integer copy number;

ii) with two variants of different copy number, but no selection i.e. *s_i_* =0 for all *i*;

iii) with two variants of different copy number, one of which had a selective advantage or disadvantage relative to the other.

In combined models of chromosomal copy number change, each variant was characterized by the copy number at two different chromosomes, giving four genetic variants.

Given its parameters, our model generates expected variant frequencies *x_i_*(*t_k_*) for each variant and time-point considered. These were compared to the observed variant frequencies *n_i_(t_k_)/N(t_k_)*, where *n_i_(t_k_)* is the number of times a variant was observed in a population at *t_k_*, and N(t_k_) is the total number of observations at this time. Comparison was performed using a likelihood framework. Given data describing allele frequencies, the likelihood was calculated using a multinomial framework:

Evaluation of chromosomal copy number data was conducted using a double Poisson distribution. The double Poisson distribution allows for the evaluation of count data from a distribution that may be over-dispersed, or under-dispersed, relative to a standard Possion distribution, having two parameters, *μ* and *θ*, which characterize the mean (approximately equal to *μ*) and the variance (approximately equal to *μ*/*θ*) of the distribution (Efron, 1986). In order to obtain a conservative estimate of the variance in the chromosomal copy number data, we calculated the optimal value of *θ* under the assumption that the underlying chromosomal copy numbers did not change over time, all observed changes resulting from noise in the data.

Chromosomal copy numbers were reported relative to the underlying whole-genome copy number for a diploid region (i.e. a value of 1 indicates diploidy). Considering these values, we calculated the mean of this statistic for each chromosome *c*, as:

where *n_c_(t_k_)* was the observed copy number of *c* at time *t_k_*, and *n_g_(t_k_)* was the observed genome-wide copy number at time *t_k_*. We then defined the double Poisson likelihood for the time-resolved samples from a chromosome:

where the function *C* is approximated by the relation:

Using optimization routines within the Mathematica software package, we then identified the value of theta maximizing the total likelihood:

This calculation gave the value *θ*=3.4447, indicating substantial under-dispersion relative to a Poisson model. The resulting model was used for evaluating models of chromosome copy number change.

A two-chromosome copy number model was fitted to look for an interaction between the chromosome copy number changes in chromosome 9 and those in chromosomes 31 and 33. In each case, although a model of selection acting at two loci gave a good visual fit to the dip in copy number in chromosomes 31 and 33, there was not sufficient evidence to support either two-way interaction, the optimal model in each case being one of selection for the copy number change in chromosome 9 alone.

In cases where a change in allele frequency or copy number occurred within the space of a single time point, it was not possible to estimate the strength of selection in a meaningful way, other than to say that it was large.

**Table S1**. Illumina Hiseq DNA read sequence output for each sample. Drug, MIL concentration (µM) point at which DNA was extracted; WT, Wild Type; Passages since isolation from the patient (since cloning from the parental isolate); Depth, Median read depth

| **WHO name** | **Sanger ID** | **Drug** | **Passages** | **Depth** | **Accession numbers** |
| --- | --- | --- | --- | --- | --- |
| BPK275/0cl18 WT | BPK275A1_3 | WT | R51(20) | 26 | ERS197371 |
| BPK275/0cl18 | BPK275A1M4 | WT | R83(52) | 88 | ERS160174 |
| BPK275/0cl18_SCMIL1.25 | BPK275A1Ma | 3 | R51(19) | 112 | ERS106918 |
| BPK275/0cl18_SCMIL2.5 | BPK275A1Mb | 6 | R51(19) | 61 | ERS106934 |
| BPK275/0cl18_SCMIL5 | BPK275A1Mc | 12 | R57(25) | 53 | ERS106920 |
| BPK275/0cl18_SCMIL10 | BPK275A1Md | 35 | R58(26) | 68 | ERS106921 |
| BPK275/0cl18_SCMIL20 | BPK275A1Me | 49 | R66(34) | 68 | ERS106935 |
| BPK275/0cl18_SCMIL25 | BPK275A1Mf | 61 | R71(39) | 76 | ERS106923 |
| BPK275/0cl18_SCMIL30subclone9meta1 | BPK275F6M1 | 74 | R91(58) | 57 | ERS067622 |
| BPK275/0cl18_SCMIL30subclone9meta2 | BPK275F6M2 | 74 |  | 64 | ERS067624 |
| BPK275/0cl18_SCMIL30subclone9meta3 | BPK275F6M3 | 74 |  | 68 | ERS067632 |
| BPK275/0cl18_SCMIL30clone9meta4 | BPK275F6M4 | 74 |  | 68 | ERS074330 |
| BPK275/0cl18_SC MIL30_sc9 | BPK275F6M5 | 74 |  | 30 | ERS160175 |
| BPK275/0cl18_SCMIL30_scl9 | BPK275F6M5_1 | 74 | R116 (83) | 66 | ERS197374 |
| BPK275/0cl18_SCMIL30 subclone9_MIL_NEG3 | BPK275F6MNEG | 74+0 |  | 43 | ERS159469 |
| BPK275/0cl18_SCMIL30 subclone9_MILPOS | BPK275F6MPOS | 74+74 |  | 26 | ERS159468 |
| BPK275/0cl18_SCMIL30cl9MILNEG2 | BPK275F6N2 | 74+0 |  | 88 | ERS106929 |
| **WHO name** | **Sanger ID** | **Drug** | **Passages** | **Depth** | **Accession numbers** |
| BPK282/0cl4WT | BPK282A1_1 | WT | R41(20) | 27 | ERS197372 |
| BPK282/0cl4WTmeta1 | BPK282A1M1 | WT |  | 63 | ERS067627 |
| BPK282/0cl4WTmeta2 | BPK282A1M2 | WT |  | 26 | ERS074325 |
| BPK282/0cl4 | BPK282A1M3 | WT |  | 21 | ERS160170 |
| BPK282/0cl4_SCMIL1.25 | BPK282A1Ma | 3 | R41(18) | 45 | ERS106906 |
| BPK282/0cl4_SCMIL2.5 | BPK282A1Mb | 6 | R41(18) | 24 | ERS106938 |
| BPK282/0cl4_SCMIL5 | BPK282A1Mc | 12 | R50(27) | 40 | ERS106908 |
| BPK282/0cl4_SCMIL10 | BPK282A1Md | 35 | R50(27) | 43 | ERS106909 |
| BPK282/0cl4_SCMIL20 | BPK282A1Me | 49 | R58(35) | 92 | ERS106931 |
| BPK282/0cl4_SCMIL25 | BPK282A1Mf | 61 | R63(40) | 68 | ERS106911 |
| BPK282/0cl4_SCMIL30subclone8meta1 | BPK282O15M1 | 74 | R67(57) | 57 | ERS067628 |
| BPK282/0cl4_SCMIL30subclone8meta2 | BPK282O15M2 | 74 |  | 96 | ERS074326 |
| BPK282/0cl4_SCMIL30 subclone 8 | BPK282O15M3 | 74 |  | 28 | ERS160171 |
| BPK282/0cl4_SCMIL30_scl8 | BPK282O15M3_1 | 74 | R91 (76) | 68 | ERS197375 |
| BPK282/0cl4_SCMIL30 subclone8MILNEG1 | BPK282O15N1 | 74+0 |  |  |  |

**Table S2.** Chromosome copy numbers in BPK282/0cl4 (Sb-S) and BPK275/0cl18 (Sb-R), before and after the MIL-R selection (respectively WT and MIL-R).

|  | Observed | | | |  |  | Differences | | | |  | Result |
| --- | --- | --- | --- | --- | --- | --- | --- | --- | --- | --- | --- | --- |
|  | **WT** | | **MIL-R** | |  |  | **Sb-S vs Sb-R** | | **MIL-S vs MIL-R** | |  |  |
| Chr | **Sb-S** | **Sb-R** | **Sb-S** | **Sb-R** |  | **Chr** | **WT** | **MIL-R** | **Sb-S** | **Sb-R** |  |  |
| 1 | 1.96 | 1.96 | 1.96 | 2.00 |  | 1 | 0.00 | 0.00 | 0.00 | 0.04 |  | No change |
| 2 | 2.08 | 4.12 | 2.08 | 3.18 |  | 2 | 2.04 | 1.10 | 0.00 | -0.94 |  | Down in Sb-R MIL-R |
| 3 | 2.08 | 2.00 | 2.00 | 2.10 |  | 3 | -0.08 | 0.10 | -0.08 | 0.10 |  | No change |
| 4 | 2.04 | 1.96 | 2.00 | 2.00 |  | 4 | -0.08 | 0.00 | 0.00 | 0.04 |  | No change |
| 5 | 3.06 | 1.96 | 3.00 | 2.10 |  | 5 | -1.10 | -0.90 | -0.06 | 0.14 |  | WT differences remain |
| 6 | 2.24 | 2.04 | 3.08 | 2.10 |  | 6 | -0.20 | -0.98 | 0.84 | 0.06 |  | Down in Sb-R MIL-R |
| 7 | 2.12 | 2.04 | 2.04 | 2.10 |  | 7 | -0.08 | 0.06 | -0.08 | 0.06 |  | No change |
| 8 | 2.48 | 3.92 | 2.04 | 3.00 |  | 8 | 1.44 | 0.96 | -0.44 | -0.92 |  | Down in both MIL-R |
| 9 | 3.10 | 2.98 | 3.12 | 4.10 |  | 9 | -0.12 | 0.98 | 0.00 | 1.12 |  | Up in Sb-R MIL-R |
| 10 | 2.04 | 2.00 | 2.04 | 2.10 |  | 10 | 0.00 | 0.06 | 0.00 | 0.10 |  | No change |
| 11 | 2.12 | 3.10 | 2.08 | 3.18 |  | 11 | 0.98 | 1.10 | 0.00 | 0.08 |  | WT differences remain |
| 12 | 2.12 | 2.08 | 2.08 | 2.10 |  | 12 | 0.00 | 0.00 | 0.00 | 0.02 |  | No change |
| 13 | 2.80 | 2.98 | 2.04 | 2.00 |  | 13 | 0.18 | 0.00 | -0.76 | -0.98 |  | Down in both MIL-R |
| 14 | 2.12 | 2.94 | 2.96 | 3.00 |  | 14 | 0.82 | 0.00 | 0.84 | 0.06 |  | Up in Sb-S MIL-R |
| 15 | 2.16 | 2.00 | 2.04 | 2.46 |  | 15 | -0.16 | 0.42 | -0.12 | 0.46 |  | No change |
| 16 | 3.14 | 3.06 | 3.08 | 3.10 |  | 16 | -0.08 | 0.00 | -0.06 | 0.04 |  | No change |
| 17 | 2.08 | 2.04 | 2.08 | 2.10 |  | 17 | 0.00 | 0.00 | 0.00 | 0.06 |  | No change |
| 18 | 2.08 | 2.00 | 2.04 | 2.10 |  | 18 | -0.08 | 0.06 | 0.00 | 0.10 |  | No change |
| 19 | 2.00 | 1.96 | 2.00 | 2.00 |  | 19 | 0.00 | 0.00 | 0.00 | 0.04 |  | No change |
| 20 | 2.04 | 2.00 | 2.04 | 2.00 |  | 20 | 0.00 | 0.00 | 0.00 | 0.00 |  | No change |
| 21 | 2.04 | 2.00 | 2.04 | 2.00 |  | 21 | 0.00 | 0.00 | 0.00 | 0.00 |  | No change |
| 22 | 2.08 | 2.00 | 2.04 | 2.10 |  | 22 | -0.08 | 0.06 | 0.00 | 0.10 |  | No change |
| 23 | 3.10 | 3.02 | 4.12 | 3.10 |  | 23 | -0.08 | -1.02 | 1.02 | 0.08 |  | Up in Sb-S MIL-R |
| 24 | 2.04 | 1.96 | 2.04 | 2.00 |  | 24 | -0.08 | 0.00 | 0.00 | 0.04 |  | No change |
| 25 | 2.04 | 2.00 | 2.04 | 2.10 |  | 25 | 0.00 | 0.06 | 0.00 | 0.10 |  | No change |
| 26 | 3.02 | 2.00 | 2.70 | 2.00 |  | 26 | -1.02 | -0.70 | -0.32 | 0.00 |  | WT differences remain |
| 27 | 2.04 | 2.00 | 2.08 | 2.10 |  | 27 | 0.00 | 0.00 | 0.00 | 0.10 |  | No change |
| 28 | 2.04 | 1.96 | 2.04 | 2.00 |  | 28 | -0.08 | 0.00 | 0.00 | 0.04 |  | No change |
| 29 | 2.04 | 2.00 | 2.04 | 2.00 |  | 29 | 0.00 | 0.00 | 0.00 | 0.00 |  | No change |
| 30 | 2.08 | 2.00 | 2.08 | 2.00 |  | 30 | -0.08 | -0.08 | 0.00 | 0.00 |  | No change |
| 31 | 4.12 | 3.92 | 4.04 | 4.00 |  | 31 | -0.20 | 0.00 | -0.08 | 0.08 |  | No change |
| 32 | 2.16 | 2.00 | 2.04 | 2.00 |  | 32 | -0.16 | 0.00 | -0.12 | 0.00 |  | No change |
| 33 | 2.86 | 3.96 | 2.04 | 3.64 |  | 33 | 1.10 | 1.60 | -0.82 | -0.32 |  | Down in both MIL-R |
| 34 | 2.04 | 1.96 | 2.04 | 2.00 |  | 34 | -0.08 | 0.00 | 0.00 | 0.04 |  | No change |
| 35 | 3.06 | 2.24 | 3.04 | 2.90 |  | 35 | -0.82 | -0.14 | 0.00 | 0.66 |  | Up in Sb-R MIL-R |
| 36 | 2.04 | 1.92 | 2.04 | 2.00 |  | 36 | -0.12 | 0.00 | 0.00 | 0.08 |  | No change |

In the differences table, the Sb-S vs Sb-R comparison shows positive values for a higher copy number in Sb-R than Sb-S. Likewise, the MIL-S vs MIL-R comparison shows more positive values for MIL-S compared to MIL-R. The sole consistent change of somy was for chr13.

**Table S3**. SNPs between Sb-R WT (BPK275/0cl18) and Sb-S WT (BPK282/0cl4).

| Mutation type | Numbers | Homozygous | Heterozygous |
| --- | --- | --- | --- |
| CDS SNPs | 54 | 38 | 16 |
| Nonsynonymous SNPs | 33 | 22 | 11 |
| Synonymous SNPs | 21 | 16 | 5 |
| 5’ UTR | 29 | 20 | 9 |
| 3’ UTR | 14 | 9 | 5 |
| SNPs | 127 | 92 | 36 |

**Table S4**. Genetic variants between Sb-R WT (BPK275/0cl18) and Sb-S WT (BPK282/0cl4), including (A) non-synonymous SNPs, (B) CNVs (expressed per haploid genome) and (C) indels.

(A) SNPs:

| Chr | Position | Gene ID | Type | Gene Product | Sb-R | Sb-S |
| --- | --- | --- | --- | --- | --- | --- |
| **2** | **36464** | **LdBPK_020100** | **R3452H** | **Phosphatidylinositol 3 kinase** | **TT** | **CC** |
| 2 | 156317 | LdBPK_020310 | S15L | Conserved hypothetical protein | AG | GG |
| 2 | 207857 | LdBPK_020440 | G948D | Hypothetical protein | TT | CC |
| **4** | **337729** | **LdBPK_040850** | **A113V** | **Rhomboid protein serine peptidase Clan Sfamily S54** | **AA** | **GG** |
| 5 | 325823 | LdBPK_050890 | V372A | Conserved hypothetical protein | CC | TT |
| 8 | 284923 | LdBPK_080670 | A659V | Protein kinase | TT | CC |
| **12** | **128965** | **LdBPK_120275** | **Y1230C** | **Conserved hypothetical protein** | **GG** | **AA** |
| **16** | **690776** | **LdBPK_161760** | **Y1214C** | **Conserved hypothetical protein** | **GG** | **AA** |
| 17 | 602496 | LdBPK_171390 | R443L | Translation initiation factor | GT | GG |
| 18 | 316904 | LdBPK_180770 | R132G | Conserved hypothetical protein | GG | CC |
| 18 | 502523 | LdBPK_181200 | P363L | Conserved hypothetical protein | CC | CT |
| 19 | 30197 | LdBPK_190140 | L174S | Mitogen activated protein kinase | AG | AA |
| 23 | 255065 | LdBPK_230710 | N890S | Ubiquitin activating enzyme e1 | CC | TT |
| **24** | **26882** | **LdBPK_240130** | **A150V** | **Ankyrin/TPR repeat protein** | **TT** | **CC** |
| **25** | **702972** | **LdBPK_251890** | **A61E** | **Conserved hypothetical protein** | **TT** | **GG** |
| 29 | 161487 | LdBPK_290470 | R676H | Conserved hypothetical protein | CC | CT |
| 29 | 545079 | LdBPK_291420 | N18S | Serine/threonine protein kinase | GG | AA |
| 30 | 184542 | LdBPK_300590 | K439Q | Spliceosome associated protein | GG | TT |
| 30 | 832280 | LdBPK_302230 | Q357* | Conserved hypothetical protein | AG | GG |
| **31** | **555080** | **LdBPK_311340** | **S268P** | **Conserved hypothetical protein** | **GG** | **AA** |
| 31 | 1273424 | LdBPK_312660 | A748V | Conserved hypothetical protein | AA | GG |
| 32 | 275015 | LdBPK_320820 | R722H | Protein kinase | AA | AG |
| **32** | **357021** | **LdBPK_320990** | **V125L** | **Conserved hypothetical protein** | **CC** | **GG** |
| 32 | 584671 | LdBPK_321530 | Q48R | Conserved hypothetical protein | CC | TT |
| 33 | 293557 | LdBPK_330890 | V502A | Hypothetical protein | CC | TT |
| **33** | **1316522** | **LdBPK_333140** | **V390A** | **Conserved hypothetical protein** | **CC** | **TT** |
| 34 | 1479705 | LdBPK_343590 | N260S | Conserved hypothetical protein | AG | AA |
| 34 | 1544931 | LdBPK_343760 | A33G | Conserved hypothetical protein | CG | CC |
| 35 | 94419 | LdBPK_350370 | S359F | ATP dependent DEAD box RNA helicase | CC | CT |
| 35 | 1041163 | LdBPK_352500 | N1855S | Conserved hypothetical protein | GG | AA |
| **35** | **1773762** | **LdBPK_354470** | **A54T** | **Conserved hypothetical protein** | **AA** | **GG** |
| 36 | 463670 | LdBPK_361280 | A334E | Conserved hypothetical protein | AA | CC |
| 36 | 2455489 | LdBPK_366580 | V2397A | Phosphatidylinositol 3 kinase | GG | AG |

Genes in bold have previously been implicated in population-level antimonial resistance (Downing *et al*., 2011). This included SNPs in hypothetical genes (LdBPK_161760, Y1214C; 251P, LdBPK_211240) previously differentiated Sb-S and Sb-R clinical lines. LdBPK_161760’s protein product has high homology to coleoptericin (Pfam), which has antibacterial activity in *Allomyrina* *dichotoma* (Sagisaka *et al.,* 2001). A homozygous SNP (N890S) in a gene encoding ubiquitin activating enzyme e1 (LdBPK_230710) is associated with ubiquitin mediated proteolysis (EC: 6.3.2.19) and has previously been linked to Sb-R and the VL host phenotype (Downing et al., 2012). Pentamidine modifies ubiquitin (Nguewa *et al.,* 2005) and may also inhibit aminoacylation and translation through tRNA binding (Sun and Zhang, 2008). A homozygous SNP (A150V) in a gene encoding an ankyrin-TPR repeat protein (LdBPK_240130) attaches membrane proteins to the cell membrane and has links to Sb-R (Downing *et al.*, 2011). A homozygous SNP in a gene encoding a spliceosome associated protein (K439Q in LdBPK_300590) forms part of the spliceosome involved in mRNA processing.

(B) CNVs (per haploid genome):

| **Chr** | **Start** | **End** | **Sb-S** | **Sb-R** | **Gene ID(s)** | **Gene Product(s)** |
| --- | --- | --- | --- | --- | --- | --- |
| 7 | 417,410 | 419,605 | 3.03 | 2.5 | Noncoding |  |
| 8 | 326,352 | 334,035 | 3.59 | 4.72 | LdBPK_080760 | Amastin |
| 8 | 472,407 | 485305 | 1.59 | 2.13 | LdBPK_081130, LdBPK_081160, LdBPK_081190, LdBPK_081220 | Hypothetical protein |
|  |  |  |  |  |  | Hypothetical protein |
|  |  |  |  |  |  | Hypothetical protein |
|  |  |  |  |  |  | Hypothetical protein |
| 8 | 485,966 | 502,933 | 1.51 | 2.18 | LdBPK_081250, LdBPK_081290, LdBPK_081300 | Protein kinase |
|  |  |  |  |  |  | Beta tubulin |
|  |  |  |  |  |  | Histone deacetylase |
| 19 | 570,558 | 591,180 | 5.84 | 5.14 | LdBPK_191340 | Glycerol uptake protein |
| 21 | 645,881 | 653,293 | 0.69 | 1.4 | LdBPK_211840 | Hypothetical protein |
| 25 | 879,920 | 885,804 | 1.84 | 2.79 | LdBPK_252540, LdBPK_252550, LdBPK_020010 | Aminopeptidase P1 metallo-peptidase Clan MG Family M24 |
|  |  |  |  |  |  | Tagatose-6-phosphate kinase |
|  |  |  |  |  |  | Aminopeptidase P1 metallo-peptidase Clan MG Family M24 |
| 26 | 17,025 | 25,303 | 1.77 | 0.89 | LdBPK_260080, LdBPK_260090, LdBPK_260091, LdBPK_260110, LdBPK_260120 | Hypothetical protein |
|  |  |  |  |  |  | Hypothetical protein |
|  |  |  |  |  |  | Hypothetical protein |
|  |  |  |  |  |  | Hypothetical protein |
|  |  |  |  |  |  | Adenine phosphoribosyltransferase |
| 27 | 313,378 | 316,306 | 3.79 | 3.2 | LdBPK_261000 | Dynein heavy chain |
| 27 | 1,011,415 | 1,023,760 | 4.6 | 5.42 | LdBPK_072500 | Glycosomal phosphoenolpyruvate |
| 29 | 818,174 | 827,248 | 4.27 | 3.32 | LdBPK_291880, LdBPK_291890 | Paraflagellar rod protein 1D |
|  |  |  |  |  |  | Paraflagellar rod protein 1D |
| 30 | 690,154 | 696,303 | 0.9 | 1.96 | LdBPK_301910 | Succinyl-coa:3-ketoacid-coenzyme a |
| 31 | 1,451,467 | 1,457,952 | 1.64 | 0.88 | LdBPK_313180, LdBPK_313190 | Iron/zinc transporter protein |
|  |  |  |  |  |  | Iron/zinc transporter protein |
| 31 | 1,493,968 | 1,500,660 | 1.63 | 2.24 | Noncoding |  |
| 34 | 1,162,647 | 1,166,700 | 5.12 | 4.39 | Noncoding |  |
| 36 | 2,525,414 | 2,543,755 | 14.88 | 17.09 | LdBPK_366740, LdBPK_366750, LdBPK_366760, LdBPK_366770, LdBPK_366780 | Tartrate-sensitive acid phosphatase |
|  |  |  |  |  |  | Hypothetical protein |
|  |  |  |  |  |  | Mitogen activated protein kinase |
|  |  |  |  |  |  | Histidine secretory acid phosphatase |
|  |  |  |  |  |  | Ubiquitin fusion degradation protein |

(C) Indels:

| **Chr** | **Position** | **Sb-S** | **Sb-R** | **Gene ID** | **Type** | **Gene product** |
| --- | --- | --- | --- | --- | --- | --- |
| 1 | 250,993 | 0 | -4 | LdBPK_010810 | 5' UTR indel | Hypothetical protein |
| 3 | 12,608 | 0 | -2 | LdBPK_030050 | 5' UTR indel | Hypothetical protein |
| 6 | 204,486 | 0 | -2 | LdBPK_060580 | 3' UTR indel | Deoxyuridine triphosphatase dUTP diphosphatase |
| 9 | 466,173 | 0 | 2 | LdBPK_091220 | 5' UTR indel | Hypothetical protein |
| 10 | 154,060 | -1 | -2 | Noncoding |  |  |
| 11 | 831 | 0 | 2 | LdBPK_110010 | 5' UTR indel | Hypothetical protein |
| 11 | 433,750 | 0 | 2 | LdBPK_111050 | 5' UTR indel | Pretranslocation protein alpha subunit SEC61-like |
| 12 | 181,113 | 0 | -2 | LdBPK_120380 | 5' UTR indel | Hypothetical protein |
| 13 | 543,445 | -1 | -2 | Noncoding |  |  |
| 16 | 615,093 | 0 | -2 | Noncoding |  |  |
| 18 | 41,698 | 0 | -1 | LdBPK_180140 | 3' UTR indel | Hypothetical protein |
| 18 | 256,609 | 0 | 2 | Noncoding |  |  |
| 19 | 147,468 | 0 | -2 | Noncoding |  |  |
| 19 | 184,121 | 0 | 2 | LdBPK_190460 | 3' UTR indel | Hypothetical protein |
| 19 | 276,808 | -2 | -4 | LdBPK_190610 | 3' UTR indel | Hypothetical protein |
| 19 | 326,888 | 0 | 2 | LdBPK_190710 | 5' UTR indel | Glycosomal malate dehydrogenase |
| 20 | 697,476 | 0 | -2 | LdBPK_201580 | 5' UTR indel | Cell division cycle protein |
| 23 | 61,373 | 0 | -4 | LdBPK_230220 | 3' UTR indel | Endoribonuclease L-PSP (pb5) |
| 24 | 624,088 | 0 | -2 | Noncoding |  |  |
| 24 | 809,134 | -1 | -2 | LdBPK_242200 | 5' UTR indel | Hypothetical protein |
| 25 | 239,937 | 0 | 2 | LdBPK_250700 | 5' UTR indel | Hypothetical protein |
| 26 | 521,984 | 0 | -2 | LdBPK_261450 | 5' UTR indel | Farnesyltransferase beta subunit |
| 28 | 518,204 | 1 | -2 | Noncoding |  |  |
| 28 | 795,175 | 0 | -2 | LdBPK_282120 | 5' UTR indel | Hypothetical protein |
| 28 | 822,459 | -2 | 2 | LdBPK_282170 | 5' UTR indel | NADH dehydrogenase subunit NB6M |
| 30 | 30,081 | 0 | -4 | LdBPK_300100 | 5' UTR indel | Hypothetical protein |
| 30 | 834,539 | -1 | -2 | LdBPK_302240 | 5' UTR indel | Hypothetical protein |
| 30 | 1,083,610 | 0 | -2 | LdBPK_302940 | 5' UTR indel | Hypothetical protein |
| 30 | 1,109,465 | 0 | 6 | LdBPK_303050 | 5' UTR indel | Hypothetical protein |
| 31 | 7,735 | 0 | 4 | LdBPK_310030 | 2bp deletion | Aquaglyceroporin |
| 31 | 547,695 | 0 | -2 | LdBPK_311340 | 5' UTR indel | Hypothetical protein |
| 31 | 1,045,076 | 0 | -2 | LdBPK_312170 | 3' UTR indel | Hypothetical protein |
| 32 | 291,736 | 0 | -2 | Noncoding |  |  |
| 32 | 341,598 | 0 | 4 | Noncoding |  |  |
| 32 | 934,711 | 0 | -2 | LdBPK_322520 | 5' UTR indel | Hypothetical protein |
| 32 | 1,515,277 | -1 | -2 | LdBPK_324060 | 5' UTR indel | Hypothetical protein |
| 33 | 223,829 | 0 | -2 | LdBPK_330710 | 3' UTR indel | Hypothetical protein |
| 33 | 783,927 | 0 | -2 | Noncoding |  |  |
| 33 | 856,592 | -2 | -8 | Noncoding |  |  |
| 33 | 1,372,284 | 0 | 2 | LdBPK_333210 | 5' UTR indel | Tyrosyl-DNA phosphodiesterase |
| 34 | 288,211 | -2 | -4 | Noncoding |  |  |
| 35 | 95,696 | 0 | -2 | Noncoding |  |  |
| 35 | 137,412 | 0 | 2 | Noncoding |  |  |
| 35 | 1,161,127 | -1 | -2 | Noncoding |  |  |
| 35 | 1,262,921 | -1 | -2 | Noncoding |  |  |
| 35 | 1,530,617 | -1 | -2 | Noncoding |  |  |
| 35 | 1,869,582 | -1 | -2 | LdBPK_354800 | 3' UTR indel | Hypothetical protein |
| 35 | 2,093,073 | 0 | -2 | LdBPK_355380 | 3' UTR indel | Hypothetical protein |
| 36 | 1,301,976 | -2 | -4 | LdBPK_363370 | 3' UTR indel | Hypothetical protein |
| 36 | 1,444,844 | -2 | -4 | Noncoding |  |  |
| 36 | 2,609,595 | 0 | -2 | LdBPK_366980 | 3' UTR indel | Hypothetical protein |

**Table S5**. Comparison of metabolites present in Sb-S and Sb-R WT *L. donovani* promastigotes. The mean ratio WT Sb-R/ WT Sb-S for each metabolite comes from three separate experiments, and there were 4 replicates /experiment.

| **Type of compound** | **Mean Ratio WT Sb-R/ WT Sb-S ± SD** |
| --- | --- |
| **Amino acids** |  |
| Aspartic acid | 3.3 ± 2.0 |
| Glutamic acid | 3.7 ± 0.6 |
| Histidine | 4.0 ± 0.0 |
| Homocysteine | 4.2 ± 2.7 |
| Hydantoin-5-propionic acid | 7.4 ± 3.0 |
| Isoleucine, leucine | 2.3 ± 1.0 |
| Proline | 5.0 ± 0.3 |
| Tryptophan | 3.2 ± 0.0 |
| **Hydroxy acids** |  |
| Phenyllactic acid | 0.2 ± 0.2 |
| **Purines and pyrimidines** |  |
| Hypoxanthine | 0.1 ± 0.0 |
| Xanthine | 0.5 ± 0.3 |
| **Sphingoid base** |  |
| 4-hydroxysphinganine | 5.1 ± 3.1 |
| Sphinganine (C16) | 0.2 ± 0.1 |
| **Vitamins and cofactors** |  |
| 8-amino-7-oxo-nonanoic acid | 5.4 ± 0.0 |

**Table S6**. The relative concentration of different lipids present in WT Sb-S and Sb-R *L. donovani* promastigotes parasites. The mean ratio for each lipid comes from three separate experiments for the Sb-S strain and 4 experiments for the Sb-R strain, and there were 4 replicates/experiment.

| **LPC type** | **Mean Ratio**  **WT Sb-R/WT Sb-S ± SD** | **PC type** | **Mean Ratio WT Sb-R / WT Sb-S** |
| --- | --- | --- | --- |
| LPC(18:4) | 2.6 ± 0.1 | PC(32:0) | 0.3 ± 0.0 |
| LPC(19:0) | Not detected in Sb-R strain | PC(33:0) | 0.3 ± 0.1 |
| LPC(24:0) | Not detected in Sb-R strain | PC(34:0) | 0.2 ± 0.0 |
|  |  | PC(34:1) | 0.4 ± 0.0 |
|  |  | PC(35:0) | 0.2 ± 0.0 |
|  |  | PC(35:1) | 0.4 ± 0.0 |
|  |  | PC(36:1) | 0.3 ± 0.0 |
|  |  | PC(37:1) | 0.3 ± 0.0 |
|  |  | PC(38:2) | 0.4 ± 0.0 |
|  |  | PC(38:3) | 0.5± 0.0 |
|  |  | PC(34:5) | 2.1± 0.0 |
|  |  | PC(35:5) | 1.8 ± 0.5 |
|  |  | PC(35:6) | 2.3 ± 0.6 |
|  |  | PC(36:6) | 2.1 ± 0.6 |
|  |  | PC(36:7) | 3.0 ± 0.3 |
|  |  | PC(39:8) | 1.8 ± 0.4 |
|  |  | PC(40:9) | 1.9 ± 0.5 |
|  |  | PC(40:10) | 3.4 ± 0.07 |

**Table S7.** SNPs observed ordered by MIL induction stage for Sb-S BPK282/0cl4.

| Chr^1^ | Pos^2^ | Ref^3^ | Var^4^ | Type^5^ | Gene ID^6^ | Aa^7^ or base | Gene Product Name | MIL^8^ |
| --- | --- | --- | --- | --- | --- | --- | --- | --- |
| 27 | 770742 | C | T | het | LdBPK_271870 | 5' UTR | Conserved hypothetical protein | 6 |
| 13 | 623631 | C | G | hom | LdBPK_131590 | A691P | phospholipid transporting ATPase 1 (LdMT) | 12 |
| 20 | 414213 | G | A | het | LdBPK_201010 | 5' UTR | Conserved hypothetical protein |  |
| 14^A^ | 461847 | A | C | het | LdBPK_141140 | 5' UTR | protein kinase | 49 |
| 11 | 141650 | C | G | het | LdBPK_110430 | R560 | Conserved hypothetical protein | 61 |
| 12 | 339176 | C | T | het | LdBPK_120570 | R620W | Conserved hypothetical protein |  |
| 22 | 101852 | G | A | het | LdBPK_220160 | T745 | Conserved hypothetical protein |  |
| 28 | 966341 | C | A | het | LdBPK_282610 | A216D | Vacuolar ATP synthase subunit b |  |
| 16 | 237913 | T | A | het | LdBPK_160690 | *552Y | Conserved hypothetical protein | 74 |
| 16 | 302635 | T | C | het | LdBPK_160820 | 5' UTR | Conserved hypothetical protein |  |
| 24 | 789171 | G | C | het | LdBPK_242120 | 5' UTR | Conserved hypothetical protein |  |
| 26^A^ | 652865 | C | T | het | LdBPK_261790 | F2447 | Conserved hypothetical protein |  |
| 31^B^ | 525152 | G | A | het | LdBPK_311300 | 3'UTR | ATP-binding cassette protein subfamily C (ABCC5) |  |
| 31^B^ | 730669 | C | T | het | LdBPK_311550 | 5' UTR | Conserved hypothetical protein |  |
| 32 | 273988 | G | A | het | LdBPK_320820 | A380T | protein kinase |  |
| 33 | 1404783 | C | A | het | LdBPK_333250 | S1143* | Conserved hypothetical protein |  |
| 3 | 29645 | C | T | het | LdBPK_030110 | 5' UTR | Conserved hypothetical protein | 74+0 |
| 36 | 1582259 | G | A | het | LdBPK_364290 | 5' UTR | Conserved hypothetical protein |  |

SNPs observed during MIL induction for BPK282/0cl4 ordered by MIL concentration (µM). ^1^ Chromosome. ^2^ Chromosomal position. ^3^ Reference BPK282/0cl4 allele. ^4^ Variant allele. ^5^ Homozygous or heterozygous SNP type. ^6^ Gene identifier. ^7^ Amino acid. ^8^ Miltefosine (µM) dose at which SNPs was first observed. ^A^ Trisomic, SNP affected one chromosome only. ^B^ Tetrasomic, SNP affected one chromosome only.

**Table S8.** SNPs observed ordered by MIL induction stage for Sb-R BPK275/0cl18.

| Chr^1^ | Pos^2^ | Ref^3^ | Var^4^ | Type^5^ | Gene ID^6^ | Aa^7^ or base | Gene Product Name | MIL^8^ |
| --- | --- | --- | --- | --- | --- | --- | --- | --- |
| 14^A^ | 527610 | G | C | het | LdBPK_141260 | T511 | poly(A) polymerase polynucleotide adenylyl transferase | 3 |
| 34 | 782683 | G | A | het | LdBPK_341810 | 5' UTR | Conserved hypothetical protein |  |
| 2^B^ | 300726 | G | A | hom | LdBPK_020620 | G259S | Conserved hypothetical protein | 12 |
| 17 | 602496 | G | T | hom | LdBPK_171390 | R443L | translation initiation factor |  |
| 28 | 992660 | C | T | het | LdBPK_282690 | 3' UTR | eukaryotic translation initiation factor (eIF4E) |  |
| 34 | 457304 | C | T | het | LdBPK_341070 | R542C | myosin IB heavy chain |  |
| 13 | 625111 | C | A | hom | LdBPK_131590 | E197D | phospholipid transporting ATPase 1 (LdMT) | 35 |
| 13 | 631021 | C | A | hom | LdBPK_131610 | A1153 | Conserved hypothetical protein |  |
| 13 | 569446 | C | T | hom | LdBPK_131490 | 5' UTR | EF hand protein | 49 |
| 31^B^ | 257223 | T | C | het | LdBPK_310730 | D434G | ATP dependent zinc metallo-peptidase Clan MA(E) Family M41 | 61 |
| 28 | 253748 | G | A | het | LdBPK_280700 | 5' UTR | Conserved hypothetical protein | 74 |
| 35^A^ | 1066125 | T | C | het | LdBPK_352580 | 3' UTR | Conserved hypothetical protein |  |
| 36 | 348376 | C | T | hom | LdBPK_360970 | 5' UTR | mitogen activated protein kinase |  |

SNPs observed during MIL induction for BPK275/0cl18 ordered by MIL concentration (µM). ^1^ Chromosome. ^2^ Chromosomal position. ^3^ Reference BPK282/0cl4 allele. ^4^ Variant allele. ^5^ Homozygous or heterozygous SNP type. ^6^ Gene identifier. ^7^ Amino acid. ^8^ Miltefosine (µM) dose at which SNPs was first observed. ^A^ Trisomic, SNP affected one chromosome only. ^B^ Tetrasomic, SNP affected one chromosome only.

**Table S9**. Likelihoods for alternative selection models acting on mutations.

| Variant type | Region | BIC value | | | Type of change | Strength of selection |
| --- | --- | --- | --- | --- | --- | --- |
|  |  | Single value | Neutral mutation | Selection |  |  |
| **BPK275/0cl18** |  |  |  |  |  |  |
| Somy | LdBPK_02 | 36.8 | 41.3 | 47.8 | - | - |
| Somy | LdBPK_08 | 53.4 | 54.6 | 48.0 | 4 to 3 | 0.63 |
| Somy | LdBPK_09 | 107.0 | 81.2 | 46.5 | 3 to 4 | 0.87 |
| Somy | LdBPK_13 (_a) | 131.4 | 92.2 | 42.6 | 3 to 2 | 0.92 |
| SNP | LdBPK_131590 | 359.5 | - | 11.3 | E197D | >1* |
| SNP | LdBPK_131610 | 305.2 | - | 10.9 | A1153 | >1* |
| CNV | LdBPK_272290-0440 | 29.7 | 35.6 | 41.5 | - | - |
| Somy | LdBPK_31 | 44.2 | 47.2 | 53.7 | - | - |
| CNV | LdBPK_310600-0870 | 71.8 | 76.2 | 55.7 | 4 to 3 | 0.29 |
| Somy | LdBPK_33 (_a) | 50.9 | 50.7 | 56.6 | - | - |
| Somy | LdBPK_35 | 91.3 | 60.0 | 46.0 | 2 to 3 | 0.33 |
| CNV | LdBPK_350030-0700 | 62.4 | 66.1 | 48.5 | 3 to 4 | >1* |
| CNV | LdBPK_353570-5320 | 188.6 | 130.4 | 81.1 | 2 to 3 | >1* |
| **BPK282/0cl4** |  |  |  |  |  |  |
| Somy | LdBPK_13 | 213.4 | 154.9 | 48.6 | 3 to 2 | >1* |
| CNV | LdBPK_131590 | 476.4 |  | 291.5 | Deletion | 0.00 |
| SNP | LdBPK_131590 | 476.4 |  | 291.5 | P691 | 0.12 |
| Somy | LdBPK_23 | 37.7 | 43.6 | 50.0 | - | - |
| Somy | LdBPK_33 | 158.7 | 92.6 | 47.9 | 3 to 2 | 0.19 |

Shown are Bayesian Information Criterion (BIC) values for models fitted to frequency data for SNPs, chromosome copy number variation (somy) and large copy number variants (CNVs). Models describe values obtained for the scenarios of: a single value, versus a single value with a neutral mutation, versus one with selection. The best model fit in each case had the smallest BIC. Selection coefficients are given per generation. Starred values indicate cases where a frequency change occurred within a single time points; in these cases the inference of selection is large and positive, but cannot be determined with any accuracy. For the deletion (Δ) and nonsynonymous SNP (P691) at the LdMT gene (LdBPK_131590), models of selection acting upon both variants simultaneously had a lower BIC (64.0) and larger selection strength estimates (Δ=0.17, P691=0.26), suggesting that this was a more likely model than selection on either alone.

**Table S10**. See excel

**Table S11.** Metabolites differentially regulated in MIL-R and its corresponding WT *L. donovani* promastigotes. The mean ratio MIL-R /WT for each metabolite comes from three separate experiments for the Sb-S strain and 4 experiments for the Sb-R strain, and there were 4 replicates/experiment.

| **Metabolite** | **Mean Ratio Sb-S MIL-R/ WT ± SD** | **Mean Ratio Sb-R MIL-R/ WT ± SD** |
| --- | --- | --- |
| **Amino Acid** |  |  |
| 4-Nitrophenyl-3-ketovalidamine | 2.1 ± 0.3 | 1.8 ± 0.3 |
| D-Phenylalanine | 2.0 ±.0.2 |  |
| GammaGlutamyl Glutamicacid | 2.1 ± 0.5 |  |
| Imidazol-5-yl-Pyruvate | 2.5 ± 0.7 | 1.9 ± 0.1 |
| L-Methionine | 1.9 ± 0.3 | 2.7 ±0.1 |
| L-Tyrosine | 2.6 ± 0.8 |  |
| Leu-Val | 2.3 ± 0.2 |  |
| Leucyl-leucine | 2.4 ±0.3 |  |
| Met-Ala | 3.0 ± 1.3 |  |
|  |  |  |
| **Nucleotide Metabolism** |  |  |
| Hypoxanthine | 4.6 ± 1.6 |  |
| Uridine | 2.9 ± 0.6 |  |
| Xanthine | 4.3 ± 0.3 | 1.7 ± 0.1 |
|  |  |  |
| **Biosynthesis of Secondary Metabolites** |  |  |
| Taxa-4(20),11(12)-dien-5alpha-yl acetate | 3.6 ± 0.0 |  |
|  |  |  |
| **Carbohydrate Metabolism** |  |  |
| Ala-Met-Trp-Asp | 2.5 ± 0.4 |  |
|  |  |  |
| **Lipid Metabolism** |  |  |
| Choline | 2.0 ± 0.7 |  |
| Choline phosphate | 1.9 ± 0.3 | 1.7 ± 0.2 |
| Ethanolamine phosphate |  | 0.2 ± 0.0 |
| N-methylethanolamine phosphate | Not found | 8.6 ± 0.2 |
| Phosphodimethyl ethanolamine | 3.2 ± 1.3 | 2.0 ± 0.5 |
| Glycero-3-phosphate | 2.7 ± 0.7 |  |
| Glycero-3-Phospho-1-inositol | 2.1 ± 0.2 |  |
| Glycero-3-phosphocholine | 1.8 ± 0.3 | 0.7 ± 0.3 |
| Stearoylglycerone phosphate | 3.6 ± 0.8 | 1.9 ± 0.1 |
|  |  |  |
| **Unknown function** |  |  |
| Phenolphthalin | 2.3 ± 0.2 | 1.3 ± 1.3 |
| (R)-AMAA | 2.0 ± 0.0 | 0.6 ± 0.1 |

**Table S12.** The relative concentration (ratio) of different lysophosphatidylcholines present in MIL-R / WT for Sb-S and Sb-R *L. donovani* promastigotes parasites. The mean ratio MIL-R /WT for each lipid comes from three separate experiments for the Sb-S strain and four experiments for the Sb-R strain, and there were 4 replicates/experiment.

| **Sb-R MIL/WT** | | | **Sb-S MIL/WT** | | |
| --- | --- | --- | --- | --- | --- |
| **Chain length** | **Mean Ratio ± SD** | **n** | **Chain length** | **Mean Ratio ± SD** | **N** |
| LPC(16:0) | 2.4 ± 1.4 |  | LPC(16:0) | 7.4 ± 3.9 |  |
| LPC(17:0) | 2.4 ± 1.0 |  | LPC(18:0) | 7.5 ± 3.2 |  |
| LPC(18:0) | 3.1 ± 1.4 |  | LPC(19:0) | 10.5 ± 4.7 |  |
| LPC(22:5) | 0.4 ± 0.2 |  | LPC(26:0) | 4.0 ± 1.8 |  |
| LPC(22:6) | 0.5 ± 0.4 |  |  |  |  |

**Table S13.** The relative concentration (ratio) of different phosphatidylcholines present in MIL-R / WT for Sb-S and Sb-R *L. donovani* promastigotes parasites. The mean ratio MIL-R /WT for each lipid comes from three separate experiments for the Sb-S strain and four experiments for the Sb-R strain, and there were 4 replicates/experiment.

| **Sb-R MIL/WT** | | **Sb-S MIL/WT** | |
| --- | --- | --- | --- |
| **Chain length** | **Mean Ratio** | **Chain length** | **Mean Ratio** |
| PC(39:5) | 0.4 ± 0.2 | PC(32:0) | 2.6 ± 1.5 |
| PC(39:6) | 0.5 ± 0.2 | PC(32:1) | 1.6 ± 0.3 |
| PC(39:7) | 0.4 ± 0.1 | PC(33:0) | 2.6 ± 1.4 |
| PC(40:10) | 0.5 ± 0.3 | PC(35:0) | 4.1 ± 2.8 |
| PC(40:4) | 0.5 ± 0.2 | PC(35:2) | 1.3 ± 0.2 |
| PC(40:5) | 0.4 ± 0.1 | PC(36:2) | 1.7 ± 0.2 |
| PC(42:7) | 0.4 ± 0.2 | PC(36:3) | 1.5 ± 0.1 |
| PC(42:8) | 0.4 ± 0.1 | PC(40:2) | 2.3 ± 0.6 |
| PC(42:9) | 0.4 ± 0.2 | PC(40:3) | 1.7 ± 0.3 |
| PC(44:12) | 0.2 ± 0.2 | PC(42:3) | 2.1 ± 0.7 |
|  |  | PC(44:4) | 2.8 ± 0.8 |

**Table S14**. The relative concentration of different of sphingolipids in Sb-R MIL-R compared

to Sb-R WT *L. donovani* promastigotes parasites. The mean ratio MIL-R /WT for each lipid comes from three separate experiments for the Sb-S strain and four experiments for the Sb-R strain, and there were 4 replicates/experiment. Abbreviations are as follows Cer = Ceramide, SM = Sphingomyelin

| Sphingolipid | Mean ± SD |
| --- | --- |
| Cer (34:1) N-(hexadecanoyl)-sphingenine | 0.6 ± 0.1 |
| Cer (34:0) N-(hexadecanoyl)-sphinganine | 0.4 ± 0.0 |
| Cer (36:0) N-(octadecanoyl)-sphinganine | 0.4 ± 0.0 |
| Hydroxysphingenine | 0.5 ± 0.0 |
| Sphingadienine | 0.5 ± 0.4 |
| Sphinganine | 0.5 ± 0.0 |
| Sphingosine | 0.6 ± 0.2 |
| SM(32:1) | 0.5 ± 0.0 |
| SM(34:2) | 0.5 ± 0.1 |
| SM(36:1) | 0.5 ± 0.1 |
| SM(38:1) | 0.5 ± 0.0 |
| SM(40:1) | 0.5 ± 0.0 |
| SM(40:2) | 0.5 ± 0.0 |
| SM(41:2) | 0.5 ± 0.0 |
| SM(42:1) | 0.5 ± 0.0 |

**Table S15**. Predicted LdMT protein functional impacts by different mutations.

| **Mutation** | **SIFT** | **Polyphen-2** | **SNAP** | **Accuracy** |
| --- | --- | --- | --- | --- |
|  |  |  |  |  |
| This study in *L. donovani* | | | | |
| E197D | Tolerated | Probably damaging | Non-neutral | 70% |
| A691P | Tolerated | Probably damaging | Non-neutral | 78% |

Cojean et al., 2012 in *L. donovani*

| L832F | Tolerated | Probably damaging | Neutral | 89% |
| --- | --- | --- | --- | --- |

Kulshrestha et al., 2013 in *L. donovani*

| T420N | Not tolerated | Probably damaging | Non-neutral | 78% |
| --- | --- | --- | --- | --- |
| V176D | Not tolerated | Possibly damaging | Non-neutral | 82% |

Perez-Victoria et al., 2003/2006 in *L. donovani*

| R145* |  |  |  |  |
| --- | --- | --- | --- | --- |
| W210* |  |  |  |  |
| G276V | Not tolerated | Probably damaging | Non-neutral | 82% |
| L366P | Not tolerated | Probably damaging | Non-neutral | 78% |
| F414S | Tolerated | Probably damaging | Non-neutral | 63% |
| T420N | Not tolerated | Probably damaging | Non-neutral | 78% |
| F430S | Not tolerated | Probably damaging | Non-neutral | 70% |
| A653V | Not tolerated | Probably damaging | Non-neutral | 78% |
| L780P | Not tolerated | Probably damaging | Non-neutral | 58% |
| G824D | Not tolerated | Probably damaging | Non-neutral | 87% |
| L856P | Not tolerated | Probably damaging | Non-neutral | 70% |
| I914T | Not tolerated | Possibly damaging | Non-neutral | 58% |

Coelho et al 2012 in *L. major*

| M547Δ |  |  |  |  |
| --- | --- | --- | --- | --- |
| G565R | Not tolerated | Probably damaging | Non-neutral | 87% |
| W617* |  |  |  |  |
| P782T | Not tolerated | Probably damaging | Non-neutral | 70% |
| G852D | Not tolerated | Probably damaging | Non-neutral | 70% |
| W895* |  |  |  |  |

The SIFT Seq Rep in each case was equal to one: this indicated the predictions had high support. The Polyphen-2 score indicated high confidence (value>0.97) in each case except I914T and V176D (both 0.78). The percentage confidence for the SNAP predictions is shown (Accuracy).

**Table S16.** Predicted LdMT protein surface accessibility.

| Mutation | E/B | Surface access | Z-fit score | Amino acid property changes | P(alpha helix) | P(beta strand) | P(coil) |  |
| --- | --- | --- | --- | --- | --- | --- | --- | --- |
| This study in *L. donovani* | | | | | | | | |
| E197D | E | 0.34 | -0.93 | Smaller | 0.02 | 0.28 | 0.70 |  |
| A691P | B | 0.02 | 0.37 | Hydrophilic | 0.97 | 0.00 | 0.03 |  |
|  | | | | | | | | |
| Cojean et al 2012 in *L. donovani* | | | | | | | | |
| L832F | E | 0.31 | -0.30 | Not aliphatic, aromatic gain | 0.83 | 0.04 | 0.13 |  |
|  |  |  |  |  |  |  |  |  |
| Kulshrestha et al 2013 in *L. donovani* | | | | | | | |  |
| T420N | E | 0.32 | -0.42 | Hydrophilic | 0.02 | 0.14 | 0.84 |  |
| V176D | B | 0.05 | 0.50 | Not aliphatic, negatively charged | 0.00 | 0.96 | 0.04 |  |
|  |  |  |  |  |  |  |  |  |
| Perez-Victoria et al 2003/2006 in *L. donovani* | | | | | | | | |
| R145* | E | 0.38 | -0.46 | Stop codon | 0.00 | 0.51 | 0.48 |  |
| W210* | E | 0.52 | -0.53 | Stop codon | 0.52 | 0.02 | 0.46 |  |
| G276V | B | 0.10 | -0.75 | Aliphatic, small rather than tiny | 0.06 | 0.14 | 0.80 |  |
| L366P | B | 0.05 | 0.64 | Not aliphatic, hydrophilic, small | 0.86 | 0.00 | 0.14 |  |
| F414S | B | 0.08 | 1.15 | Not aromatic, hydrophilic, polar, tiny | 0.00 | 0.96 | 0.04 |  |
| T420N | E | 0.32 | -0.42 | Hydrophilic | 0.02 | 0.14 | 0.84 |  |
| F430S | B | 0.07 | -0.56 | Not aromatic, hydrophilic, polar, tiny | 0.00 | 0.90 | 0.10 |  |
| A653V | B | 0.03 | 0.37 | Aliphatic, small rather than tiny | 0.02 | 0.85 | 0.14 |  |
| L780P | B | 0.06 | -0.54 | Not aliphatic, hydrophilic, small | 0.00 | 0.14 | 0.86 |  |
| G824D | B | 0.30 | -1.29 | Negatively charged, hydrophilic, small rather than tiny | 0.12 | 0.15 | 0.73 |  |
| L856P | B | 0.04 | 0.36 | Not aliphatic, hydrophilic, small | 0.94 | 0.01 | 0.06 |  |
| I914T | B | 0.11 | -0.75 | Not aliphatic, polar | 0.52 | 0.02 | 0.46 |  |
|  |  |  |  |  |  |  |  |  |
| Coelho et al 2012 in *L. major* | | | | | | | | |
| M547Δ | B | 0.04 | -0.71 | Deleted | 0.00 | 0.90 | 0.10 |  |
| G565R | E | 0.24 | -1.54 | Hydrophilic, bigger, polar, positive charge | 0.02 | 0.09 | 0.89 |  |
| W617* | B | 0.11 | -0.96 | Stop codon | 0.62 | 0.01 | 0.36 |  |
| P782T | B | 0.20 | -0.35 | Polar, hydrophobic | 0.69 | 0.00 | 0.30 |  |
| G852D | B | 0.14 | -0.92 | Hydrophilic, polar, negative charge | 0.56 | 0.05 | 0.39 |  |
| W895* | B | 0.10 | -0.20 | Stop codon | 0.97 | 0.00 | 0.03 |  |

Predicted LdMT protein surface accessibility was determined with NetSurfP (Petersen et al 2009). E stands for exposed at the protein surface and B for beneath surface. Hydrophobicity was determined with Membrane Protein Explorer (MPEx v3.2). The most probable secondary structure in which the amino acid is located is underlined (alpha helix or beta strand or coil).

**Table S17.** Predicted transmembrane segments of the LdMT protein.

| MPEx | TMPred | TMHMM |
| --- | --- | --- |
| 59-77 | 58-79 |  |
| 85-120 | 82-120 |  |
| 255-273 | 259-282 |  |
| 299-317 | 303-324 | 302-325 |
| 352-381 | 351-370 | 345-368 |
| 462-480 |  |  |
| 662-680 | 662-680 |  |
| 765-783 |  |  |
| 850-868 |  |  |
| 874-892 | 871-892 | 869-892 |
| 897-915 | 897-915 | 896-915 |
| 942-970 | 948-968 | 944-964 |
| 990-1082 | 977-1000 | 976-999 |
|  | 1010-1030 | 1008-1031 |
|  | 1052-1073 | 1051-1074 |

Predicted transmembrane portions of the LdMT protein using Membrane Protein Explorer (MPEx v3.2), TMPred and TMHMM v2.0. MPEx predicted 13 TM helical regions compared to 12 for TMPred and 8 for TMHMM: 7 were consistent among all three tools, and only mutation P782T in *L. major* was definitely in a TM region.

**Fig S1.** Modelled allele frequency changes at the LdMT gene.


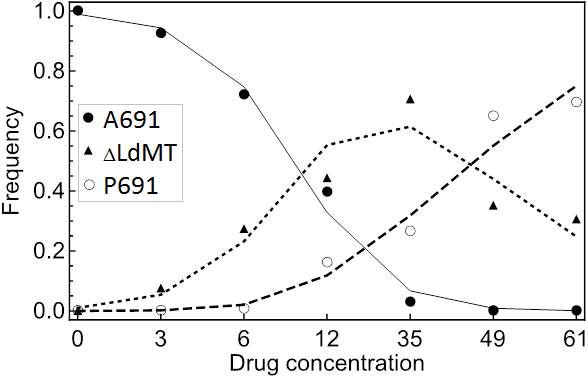


Observed and inferred frequencies for allele frequency changes (y-axis) at the LdMT gene (LdBPK_131590) in response to the drug (μM). Observed frequencies are shown for the A691, ΔLdMT, and P691 variants as solid circles, triangles, and open circles respectively. Corresponding inferences are shown as solid, dotted, and dashed lines respectively.

**References**

Coelho AC, Boisvert S, Mukherjee A, Leprohon P, Corbeil J, Ouellette M. Multiple mutations in heterogeneous miltefosine-resistant *Leishmania major* population as determined by whole genome sequencing. *PLoS Negl Trop Dis.* 2012; **6:** e1512.

Cojean S, Houzé S, Haouchine D, Huteau F, Lariven S, Hubert V, Michard F, Bories C, Pratlong F, Le Bras J, Loiseau PM, Matheron S. *Leishmania* resistance to miltefosine associated with genetic marker. *Emerg Infect Dis.* 2012; **18:** 704-706

Davletov BA, Südhof TC. A single C2 domain from synaptotagmin I is sufficient for high affinity Ca2+/phospholipid binding. *J Biol Chem.* 1993; **268:** 26386-26390.

Downing T, Imamura H, Decuypere S, Clark TG, Coombs GH, Cotton JA, Hilley JD, de Doncker S, Maes I, Mottram JC, Quail MA, Rijal S, Sanders M, Schönian G, Stark O, Sundar S, Vanaerschot M, Hertz-Fowler C, Dujardin JC, Berriman M. Whole genome sequencing of multiple *Leishmania donovani* clinical isolates provides insights into population structure and mechanisms of drug resistance. *Genome Res.* 2011; **21:** 2143-2156.

Downing T, Stark O, Vanaerschot M, Imamura H, Sanders M, Decuypere S, de Doncker S,

Maes I, Rijal S, Sundar S, Dujardin JC, Berriman M, Schönian G. Genome-wide SNP and

microsatellite variation illuminate population-level epidemiology in the *Leishmania donovani*

species complex. *Infect Genet Evol.* 2012; **12:** 149-59.

Efron B. Double exponential families and their use in generalized linear Regression. *J. American Statistical Association,* 1986; **8:** 709-721.

Hoffmann MM, Stoffel W. Construction and functional characterization of recombinant

fusion proteins of human lipoprotein lipase and apolipoprotein CII. *Eur J Biochem.* 1996;

**237:** 545-52.

Illingworth CJR, Mustonen V. A method to infer positive selection from marker dynamics in an asexual population. *Bioinformatics* 2012; **28**: 831-837.

Krogh A, Larsson B, von Heijne G, Sonnhammer EL. Predicting transmembrane protein

topology with a hidden Markov model: application to complete genomes. *J Mol Biol.* 2001;

**305:** 567-80.

Kulshrestha A, Sharma V, Singh R, Salotra P. Comparative transcript expression analysis of miltefosine-sensitive and miltefosine-resistant *Leishmania donovani*. *Parasitol Res.* 2014; **113:** 1171-1184.

Kyte J, Doolittle RF. A simple method for displaying the hydropathic character of a protein. *J*

*Mol Biol.* 1982; **157:** 105-32.

Nguewa PA, Fuertes MA, Cepeda V, Iborra S, Carrión J, Valladares B, Alonso C, Pérez JM.

Pentamidine is an antiparasitic and apoptotic drug that selectively modifies ubiquitin. *Chem*

*Biodivers.* 2005; **2:** 1387-400.

Notredame C, Higgins DG, Heringa J. T-Coffee: A novel method for fast andaccurate

multiple sequence alignment. *J Mol Biol.* 2000; **302:** 205-17.

Pérez-Victoria FJ, Gamarro F, Ouellette M, Castanys S. Functional cloning of the miltefosine transporter. A novel P-type phospholipid translocase from *Leishmania* involved in drug resistance. *J Biol Chem.* 2003; **278:** 49965-49971.

Pérez-Victoria FJ, Sánchez-Cañete MP, Castanys S, Gamarro F. Phospholipid translocation and miltefosine potency require both *L. donovani* miltefosine transporter and the new protein LdRos3 in *Leishmania* parasites. *J Biol Chem.* 2006; **281:** 23766-23775.

Petersen B, Petersen TN, Andersen P, Nielsen M, Lundegaard C. A generic method for assignment of reliability scores applied to solvent accessibility predictions. *BMC Struct Biol*. 2009; **9:** 51.

Sagisaka A, Miyanoshita A, Ishibashi J, Yamakawa M. Purification, characterization and gene expression of a glycine and proline-rich antibacterial protein family from larvae of a beetle, *Allomyrina dichotoma*. *Insect Mol Biol.* 2001; **10:** 293-302

Stoffel W, Düker M, Hofmann K. Molecular cloning and gene organization of the mouse

mitochondrial 3,2-trans-enoyl-CoA isomerase. *FEBS Lett.* 1993; **333:** 119-22.

Sun T, Zhang Y. Pentamidine binds to tRNA through non-specific hydrophobic interactions

and inhibits aminoacylation and translation. *Nucleic Acids Res.* 2008; **36:** 1654-64.
